# The protein kinase Ire1 impacts pathogenicity of *Candida albicans* by regulating homeostatic adaptation to endoplasmic reticulum stress

**DOI:** 10.1111/cmi.13307

**Published:** 2021-01-26

**Authors:** Shabnam Sircaik, Elvira Román, Priyanka Bapat, Keunsook K. Lee, David R. Andes, Neil A. R. Gow, Clarissa J. Nobile, Jesús Pla, Sneh Lata Panwar

**Affiliations:** ^1^ Yeast Molecular Genetics Laboratory School of Life Sciences, Jawaharlal Nehru University New Delhi India; ^2^ Departamento de Microbiología y Parasitología‐IRYCIS Facultad de Farmacia, Universidad Complutense de Madrid Madrid Spain; ^3^ Department of Molecular and Cell Biology University of California Merced California USA; ^4^ Quantitative and System Biology Graduate Program University of California Merced California USA; ^5^ The Aberdeen Fungal Group, MRC Centre for Medical Mycology School of Medicine, Medical Sciences & Nutrition, Institute of Medical Sciences, University of Aberdeen Aberdeen UK; ^6^ Department of Medicine Section of Infectious Diseases, University of Wisconsin Madison Wisconsin USA; ^7^ Medical Research Council Centre for Medical Mycology University of Exeter Exeter UK; ^8^ Health Sciences Research Institute University of California Merced California USA

## Abstract

The unfolded protein response (UPR), crucial for the maintenance of endoplasmic reticulum (ER) homeostasis, is tied to the regulation of multiple cellular processes in pathogenic fungi. Here, we show that *Candida albicans* relies on an ER‐resident protein, inositol‐requiring enzyme 1 (Ire1) for sensing ER stress and activating the UPR. Compromised Ire1 function impacts cellular processes that are dependent on functional secretory homeostasis, as inferred from transcriptional profiling. Concordantly, an Ire1‐mutant strain exhibits pleiotropic roles in ER stress response, antifungal tolerance, cell wall regulation and virulence‐related traits. Hac1 is the downstream target of *C. albicans* Ire1 as it initiates the unconventional splicing of the 19 bp intron from *HAC1* mRNA during tunicamycin‐induced ER stress. Ire1 also activates the UPR in response to perturbations in cell wall integrity and cell membrane homeostasis in a manner that does not necessitate the splicing of *HAC1* mRNA. Furthermore, the Ire1‐mutant strain is severely defective in hyphal morphogenesis and biofilm formation as well as in establishing a successful infection in vivo. Together, these findings demonstrate that *C. albicans* Ire1 functions to regulate traits that are essential for virulence and suggest its importance in responding to multiple stresses, thus integrating various stress signals to maintain ER homeostasis.

## 1 INTRODUCTION

Eukaryotic cells have evolved sophisticated mechanisms to ensure proper folding of proteins, especially the secretory and transmembrane proteins, through processes that largely occur in the endoplasmic reticulum (ER). The ER is also the site for other diverse cellular functions and is implicated in conveying signals to other organelles. Therefore, ER integrity is crucial for cell homeostasis and necessitates the requirement for adaptive regulatory mechanisms for its maintenance. The unfolded protein response (UPR) is one such adaptive mechanism that is activated in response to the presence of unfolded or misfolded proteins in the ER (Kimata & Kohno, 2011; Walter & Ron, 2011). Consequentially ‘ER stress’, when created and if it persists, can lead to metabolic and neurodegenerative disorders and can cause cancer in humans (Wang & Kaufman, 2012).

ER homeostasis facilitates the fulfilment of cellular secretory demands and goes hand in hand with the ability of pathogenic fungi to trigger the UPR during an infection in order to adapt to ER stress conditions. ER quality control operates in several pathogenic fungi where it is pivotal in modulating antifungal resistance and virulence (Krishnan & Askew, 2014). Upon sensing ER stress, fungi activate the UPR pathway resulting in the activation of a subset of genes that facilitate cells to regain ER homeostasis (Malhotra & Kaufman, 2007). The ER‐resident transmembrane protein, inositol‐requiring enzyme 1 (Ire1), functions as the ER stress sensor in fungi. In *Saccharomyces cerevisiae*, Ire1 oligomerizes via its luminal domain upon interaction with unfolded proteins (Cox, Shamu, & Walter, 1993; Mori, Ma, Gething, & Sambrook, 1993). Subsequent Ire1 autophosphorylation facilitates signalling through its cytosolic domain and activates its ribonuclease activity (Korennykh et al., 2009; Lee et al., 2008). This series of events is then followed by the splicing of an intron in the bZIP transcription factor *HAC1* by active Ire1 (Cox & Walter, 1996; Mori, Kawahara, Yoshida, Yanagi, & Yura, 1996). Functional Hac1 migrates to the nucleus and activates the expression of UPR target genes such as those encoding ER‐resident chaperones and protein‐modifying enzymes (Travers et al., 2000). The final outcome is the translocation of the misfolded protein from the ER to the cytosol for proteasome‐mediated degradation by a process referred to as ER‐associated degradation (ERAD; Ruggiano, Foresti, & Carvalho, 2014). Autophagy, an alternative degradative response mechanism, is also activated to remove the damaged organelles including the ER (Yorimitsu & Klionsky, 2007).

The activation of the UPR in pathogenic fungi such as *Aspergillus fumigatus* (Ire1‐Hac1) and *Cryptococcus neoformans* (Ire1‐Hxl1; Hac1 orthologue) occurs in response to various stressors and is initiated by Ire1‐dependent unconventional splicing of an intron in *HAC1* mRNA. Disruption of Ire1 function in these fungi results in increased susceptibility to antifungal drugs that target the cell wall or membrane and compromises their virulence (Cheon et al., 2011; Feng et al., 2011). Despite the conservation of the Ire1‐Hac1‐mediated UPR pathway in most eukaryotic species, *Candida glabrata* Ire1 functions independent of Hac1 to counter ER stress (Miyazaki, Nakayama, Nagayoshi, Kakeya, & Kohno, 2013). This indicates that Ire1‐dependent stress responsive pathways have diversified significantly in *C. glabrata*, compared to other fungi.

*Candida albicans*, a commensal and an opportunistic pathogen, is the major cause of mucosal and systemic fungal infections in individuals with compromised immune systems and individuals with dysbiosis of the microbiota (Polvi, Li, O'Meara, Leach, & Cowen, 2015). During ER stress, the *C. albicans HAC1* mRNA undergoes an unconventional splicing event to remove a 19 bp intron, similar to Hac1 homologues in other fungi. This splicing event generates a functional Hac1 that activates UPR target genes to mount an ER stress response in *C. albicans* (Wimalasena et al., 2008). As the involvement of Ire1 in this splicing event remains unexplored, we decided to characterize this key component and its contribution to the UPR in *C. albicans*. Here, we provide experimental evidence to establish that during tunicamycin‐induced ER stress, the processing of *HAC1* ^*u*^ (unspliced) mRNA is dependent on functional Ire1 and that *C. albicans* is reliant on the canonical Ire1‐Hac1‐mediated UPR pathway for ER quality control. Additionally, a comprehensive analysis of Ire1‐mutant phenotypes in this fungus revealed significant impacts of Ire1 on various physiological processes such as cell wall maintenance, antifungal resistance, hyphal morphogenesis and biofilm formation. Accumulated evidence indicates that ER stress responses are interwoven with fungal pathogenicity in most fungi (Krishnan & Askew, 2014). In this study, we show that *C. albicans* also relies on Ire1‐mediated stress responses for its pathogenesis, thus expanding the link between Ire1‐dependent stress responsive pathways and fungal pathogenesis to *C. albicans*.

## 2 RESULTS

### 2.1 Ire1 activity promotes cell survival to various stresses

**FIGURE 1:**
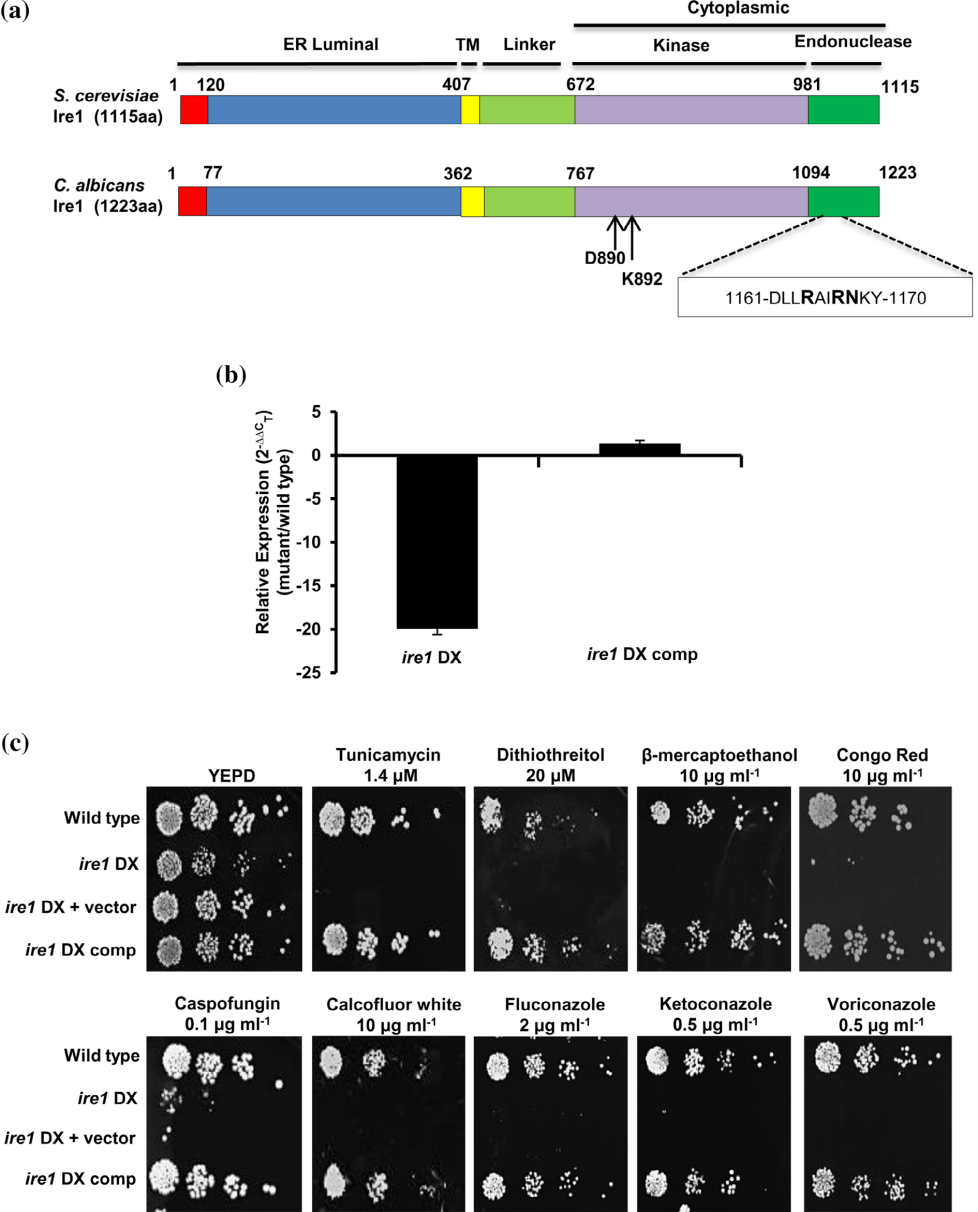
Ire1 is required for various stress responses in *C. albicans*. (a) Schematic representation of the conserved domain structure of *S. cerevisiae* and *C. albicans* Ire1. ER, endoplasmic reticulum; TM, transmembrane. Arrows indicate the conserved catalytic residues in the nucleotide‐binding pocket of the Ire1 kinase domain. The box indicates 10 amino acid residues with the three highly conserved amino acid residues (indicated in bold) in the active site of the Ire1 endonuclease domain. (b) qPCR analysis for expression of *IRE1* in the indicated strains. Fold change is calculated by 2^−ΔΔC^_T_, normalized to *ACT1* (endogenous control). Values are mean ± *SD* derived from three independent RNA preparations. (c) For phenotypic comparison, fivefold serial dilutions of cell suspensions of indicated strains were spotted on YEPD plates supplemented with drugs at specified concentrations. Plates were incubated at 30°C for 48 hr

Given that the protein structure of Ire1 is most conserved in eukaryotes including fungal members, we first analysed the *C. albicans* Ire1 for sequence conservation with other fungal Ire1 orthologues. For this, we aligned Ire1 amino acid sequences of *C. albicans*, *Ca*Ire1 (NCBI Accession No: AOW26437); *S. cerevisiae*, *Sc*Ire1 (NCBI Accession No: DAA06773); *C. glabrata*, *Cg*Ire1 (NCBI Accession No: CAG59035); *A. fumigatus*, *Af*Ire1 (NCBI Accession No: EAL87884) and *C. neoformans*, *Cn*Ire1 (NCBI Accession No: OXH74833) using Clustal Omega (Figure S1)*. Ca*Ire1 shares 51.0% similarity and 35.2% identity with *Sc*Ire1, and 46.3% similarity and 30.1% identity with *Cg*Ire1 (EMBOSS Needle; Figure S1). Sequence alignment results suggest that the core domain structure of Ire1 is conserved among these fungal species. *Ca*Ire1 consists of an N‐terminal hydrophobic signal sequence followed by an ER‐luminal domain, a transmembrane domain, a serine‐threonine protein kinase catalytic domain and a nuclease domain at the C‐terminus; these domains are also conserved in Ire1 orthologues (Figure 1a). The signal sequence at the N‐terminus and the ER luminal domain serve as determinants for the localization of Ire1 to the ER membrane and for the sensing of unfolded proteins, respectively (Credle, Finer‐Moore, Papa, Stroud, & Walter, 2005; Gardner & Walter, 2011). The transmembrane domain tethers Ire1 to the ER membrane and is shown to play a key role in sensing alterations in membrane lipid composition (Halbleib et al., 2017; Tam et al., 2018). The serine‐threonine protein kinase catalytic domain is the most conserved segment of Ire1 that allows for its autophosphorylation concomitant with the induction of its nuclease activity (Papa, Zhang, Shokat, & Walter, 2003; Shamu & Walter, 1996; Sidrauski & Walter, 1997).

Ire1 is classified as an essential gene based on a prior study that was unable to obtain a null mutant of this gene in *C. albicans* (Blankenship, Fanning, Hamaker, & Mitchell, 2010). Therefore, an *ire1* diminished expression (*ire1* DX) mutant strain was constructed by deleting one allele of *IRE1* and replacing the 5′ region of the second allele with the weakly expressed *PGA5* promoter (Woolford et al., 2016). We generated an *ire1* DX complemented strain (*ire1* DX comp) by introducing a wild‐type *IRE1* allele at the *HIS1* locus in the *ire1* DX mutant strain background (Table S1, see Materials and Methods). RT‐PCR and qPCR analyses on the *ire1* DX mutant strain confirmed diminished expression of *IRE1*, which was restored to wild‐type levels in the *ire1* DX comp strain (Figure 1b and Figure S2A), confirming successful reconstitution of *IRE1* expression.

Next, we analysed the ability of the *ire1* DX cells to grow in the presence of various stressors. Compared to the wild‐type and complemented strain, the *ire1* DX mutant strain exhibited considerably increased susceptibility to the cell wall stressors Congo red and calcofluor white (CFW); the ER stressors tunicamycin, DTT and 2‐mercaptoethanol (β‐ME) and the azole antifungal drugs fluconazole and ketoconazole (Figure 1c). We infer that Ire1 is essential for *C. albicans* growth in the presence of different stressors. Thereafter, the ability of various stressors to transcriptionally activate *C. albicans IRE1* was analysed. In *S. cerevisiae*, the UPR coordinates the synthesis of proteins as well as lipid components of the ER to ensure proper biogenesis of this organelle. While the synthesis of ER‐resident proteins is regulated by the UPR, membrane lipid synthesis is regulated by inositol; both inositol depletion and accumulation of misfolded proteins activate *S. cerevisiae* Ire1 (Cox, Chapman, & Walter, 1997). Therefore, in addition to the ER stressor (tunicamycin), the cell wall stressor (CFW) and the azole antifungal drug (fluconazole), we also included growth medium depleted in inositol (YNB without inositol) for analysing *IRE1* mRNA levels. Wild‐type *C. albicans* cells treated with these stressors for 1 and 5 hr did not exhibit an increase in *IRE1* mRNA levels, compared to the untreated wild type (Figure S2B). This indicates that stress‐dependent activation of Ire1 may largely be dependent on post‐transcriptional/translational modifications, in turn affecting its oligomeric status, which is a prerequisite for stimulating its kinase and RNase activities.

### 2.2 Transcriptional response to compromised Ire1 function

**FIGURE 2:**
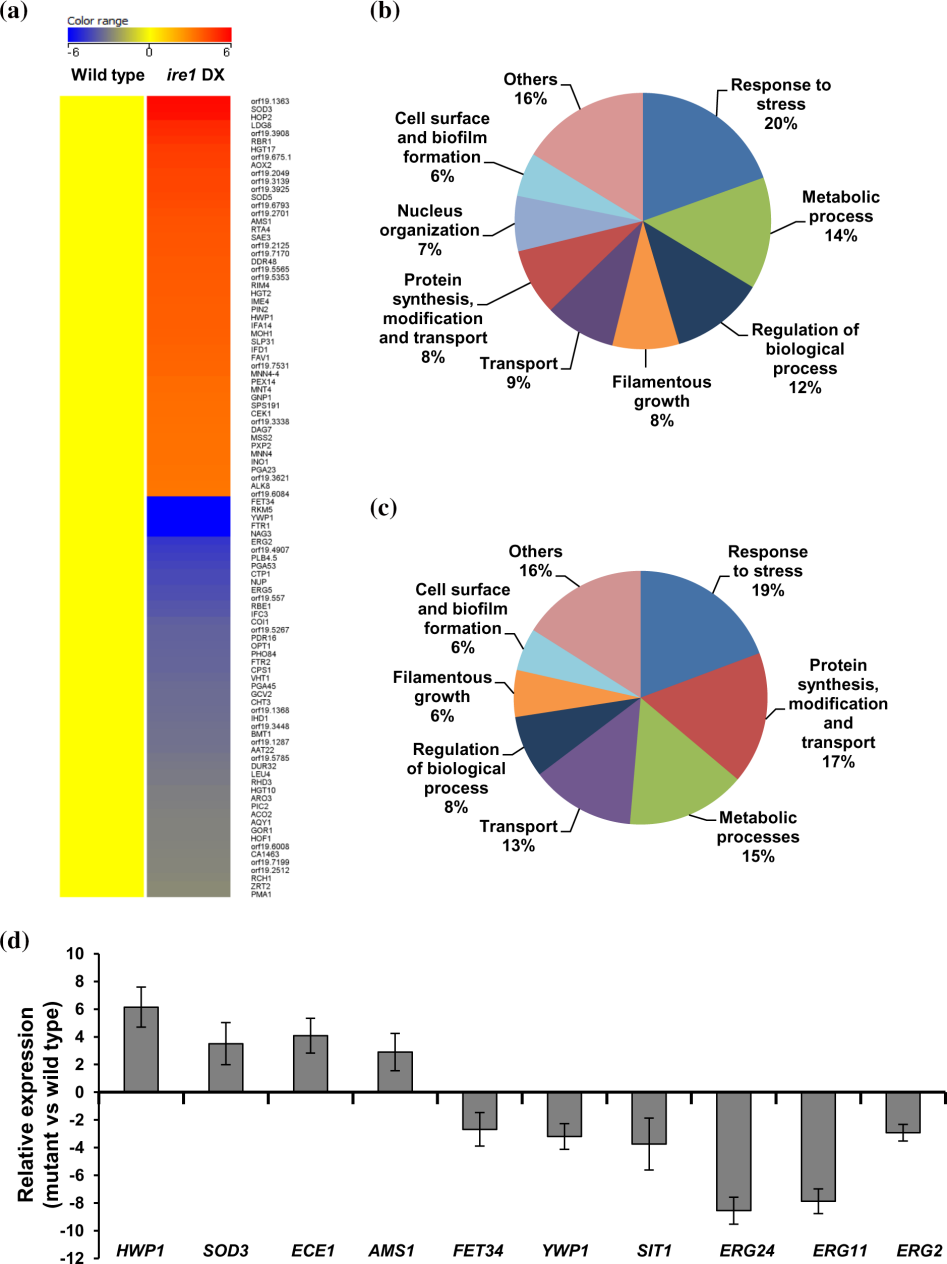
Genome‐wide transcriptional profiling of the *ire1* DXmutant strain. (a) Heat map displaying the top 50 upregulated and downregulated genes with expression fold changes of more than 1.5 and less than −1.5 with *p* value ≤.05 in *ire1* DXmutant strain versus wild type. Functional categories are represented as the percentage of total genes that were (b) downregulated and (c) upregulated in *ire1* DXmutant strain. (d) Validation of the genome‐wide transcriptional data by qPCR in the *ire1* DX mutant strain. Fold change is calculated by 2^−ΔΔC^_T_, normalized to *ACT1* (endogenous control). Values are mean ± *SD* derived from three independent RNA preparations

In order to determine the global consequences of compromised Ire1 expression in *C. albicans*, we compared the mRNA profiles of wild‐type and *ire1* DX cells grown in YEPD medium. A total of 228 genes were downregulated and 362 genes were upregulated with expression fold changes of more than 1.5 and less than −1.5 with *p* values ≤.05 in the *ire1* DX mutant strain compared to the wild‐type strain. The most conserved evident role of Ire1 is its involvement in the ER stress‐induced activation of the UPR pathway in various fungi (Feng et al., 2011; Jung, So, & Bahn, 2016; Richie et al., 2011). Given the increased susceptibility of the *ire1* DX mutant strain to tunicamycin, considered to be an indicator of ER stress (Figure 1c), we implicate *C. albicans* Ire1 in maintaining ER homeostasis plausibly by activating the UPR pathway. We therefore reasoned that cellular processes dependent upon full activation of the UPR pathway could be affected in the *ire1* DX mutant strain. In many fungi, cell wall modulation, synthesis of secretory and transmembrane proteins, translation and ribosome biogenesis are reported to be controlled by the ability of the ER to support secretory homeostasis (Feng et al., 2011; Tanaka et al., 2018; Wimalasena et al., 2008). Consistently, genes that encode for transporters such as *FET34*, *FTR1*, *NAG3*, *CTP1* and *NUP* (Figure 2a) and those that are related to protein synthesis, transport, translation and ribosome biogenesis (*RPL27A*, *RPL6*, *RPL42*, *ECM39* and *ZUO1*) were among the list of downregulated genes. *FET34* and *FTR1* are among the highest downregulated genes that are shown to be activated in iron‐limiting conditions. Both these genes code for the multicopper ferroxidases that constitute a part of the high affinity iron uptake system and contribute to hyphal morphogenesis and virulence (Chen, Pande, French, Tuch, & Noble, 2011; Cheng et al., 2013). As perturbation in secretory homeostasis also affects cell wall biogenesis and virulence traits such as hyphal morphogenesis and biofilm formation (Wimalasena et al., 2008), genes associated with these functional categories (*NAG3*, *ERG2*, *FET34* and *FTR1*, *YWP1* and *RBE1*) also featured prominently in the top 50 downregulated genes.

Gene ontology (GO) enrichment analysis identified 33 GO terms that were overrepresented for the downregulated genes in this analysis. The five highest categories of genes that were downregulated included gene classes associated with response to chemicals and stress (20%), metabolic processes (14%), regulation of biological processes (12%), transporters (9%) and protein synthesis, transport, translation and ribosome biogenesis (8%). Other significant enriched GO terms were those involved in cell surface and biofilm formation (6%), nucleus organization (7%) and filamentous growth (8%; Figure 2b).

The top 50 upregulated gene list comprised of 30 genes that are uncharaterized with unknown functions (Figure 2a). The topmost upregulated gene (C2_09880C; orf19.1363) is also an uncharacterized gene predicted to be induced under weak acid stress and during biofilm formation (Nobile et al., 2012; Ramsdale et al., 2008). The most enriched GO term out of a total of 35 tems in the upregulated gene set was cellular response to chemical/stress/drug (19%; Figure 2c). The other enriched terms included processes such as protein synthesis (translation and ribosome biogenesis), transport and modification (17%), various metabolic processess (15%), transport (13%) and filamentation (6%), followed by cell surface and biofilm formation (6%; Figure 2c). Several of the proteins encoded by the top 50 upregulated genes are those that respond to oxidative, pH or nutrition stress (*SOD3*, *RBR1*, *AOX2* and *SOD5*; Figure 2a). These findings indicate that the upregulation of stress responsive genes occurs in order to compensate for ER stress generated by the compromised expression of Ire1 in *C. albicans*. Changes in transcript levels of selected genes were verified by qPCR analysis (Figure 2d).

Interestingly, levels of expression of a large number of genes (306 out of the total of 590 diffferentially regulated genes) that are described in the Candida Genome Database (CGD) as those that are induced/repressed during biofilm formation in spider medium or on rat catheters were altered in the *ire1* DX mutant strain. Aside from this, another striking observation in our profiling data was the differential regulation of genes involved in the ergosterol biosynthesis pathway (*ERG1*, *ERG11*, *ERG2*, *ERG24*, *ERG6* and *ERG4*). Collectively, these observations suggest that regulation of cellular processes such as biofilm formation and sterol homeostasis requires Ire1‐dependent maintanence of secretory homeostasis in *C. albicans*. Thus, we surmise that compromised Ire1 function evokes a transcriptional response that can counteract perturbation in secretory homeostasis. As a result, complementary signalling networks are impacted that are essential for operating cellular processes dependent on secretory homeostasis. Altogether, our transcriptional profiling data suggests that optimal Ire1 biosynthetic capacities are required for *C. albicans* to mount a functional ER homeostatic response to maintain optimum cell/ER homeostasis.

### 2.3 Ire1 mediates the unconventional splicing of *C. albicans HAC1* mRNA to activate the UPR in response to tunicamycin

**FIGURE 3:**
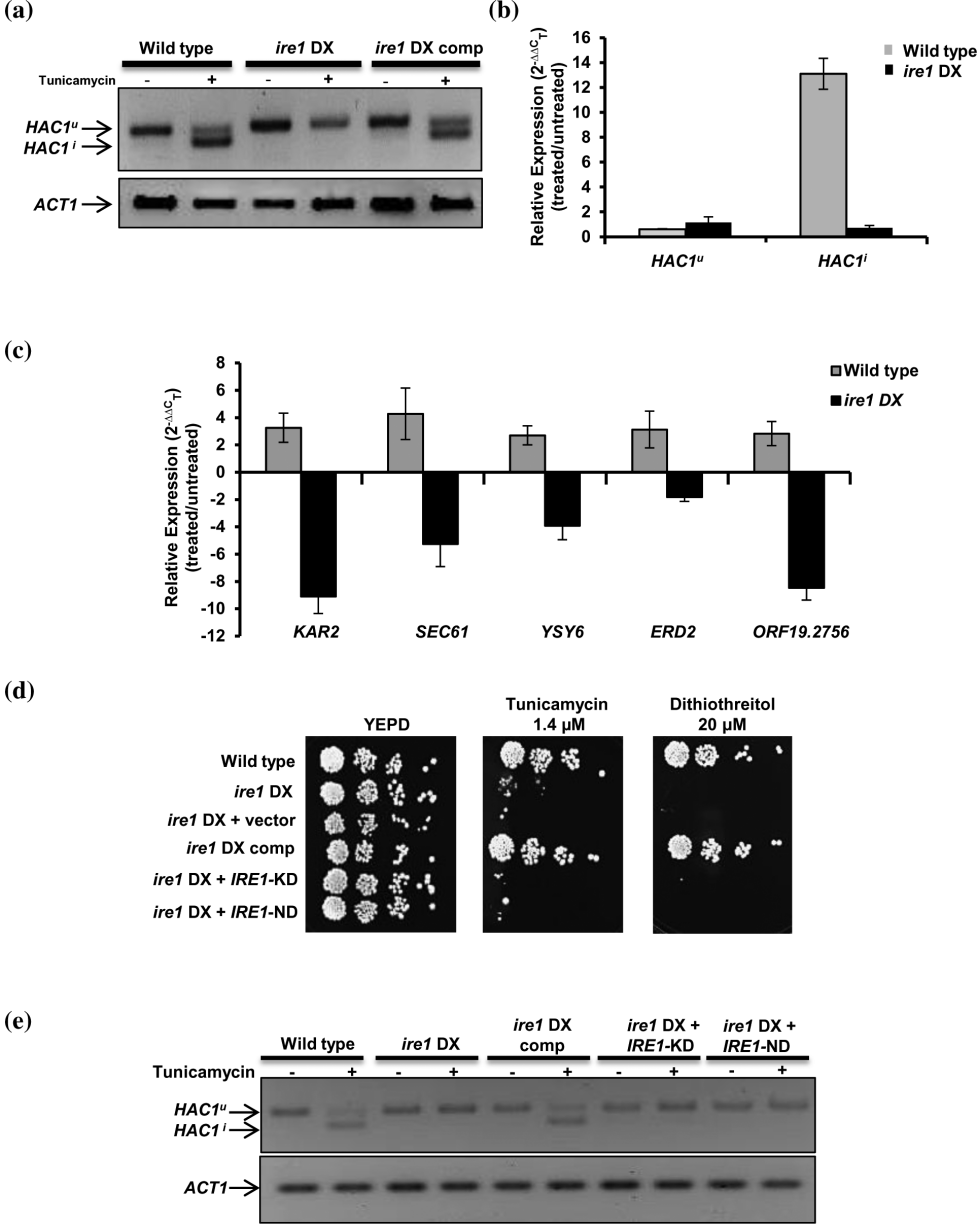
Ire1 mediates the processing of *HAC1* mRNA in response to ER stress. (a) For *HAC1* splicing pattern analysis during endoplasmic reticulum (ER) stress, cDNA was obtained from the indicated strains after treatment with 4.7 μM tunicamycin for 2 hr. PCR amplification was done using primers flanking the *HAC1* intron, and the product was analysed on a 4% agarose gel. The size difference between the spliced *HAC1* (*HAC1* ^*i*^) and unspliced *HAC1* (*HAC1* ^*u*^) isoform is 19 bp. (b) qPCR with TaqMan probe specific for spliced *HAC1* (*HAC1* ^*i*^) or unspliced *HAC1* isoform (*HAC1* ^*u*^) in wild‐type and *ire1* DXmutant strains. (c) qPCR of *HAC1*‐dependent unfolded protein response target genes in the indicated strains following tunicamycin treatment. Fold change is calculated by 2^−ΔΔC^_T_, normalized to *ACT1* (endogenous control). Values are mean ± *SD* and are derived from three independent RNA preparations. (d) For phenotypic comparison, fivefold serial dilutions of cell suspensions of the indicated strains were spotted onto YEPD plates containing indicated ER stressors. Plates were incubated at 30°C for 48 hr. (e) Analysis of *HAC1* splicing in strains containing kinase‐dead (*IRE1*‐KD) and nuclease dead (*IRE1*‐ND) version of *IRE1*. cDNA was obtained from indicated strains after treatment with 4.7 μM tunicamycin for 2 hr

Tunicamycin, by inhibiting *N*‐glycosylation, interferes with protein folding and results in the accumulation of misfolded proteins in the ER lumen (Guillemette et al., 2011). Consequentially, Ire1 binds to the unfolded proteins to activate the UPR, a conserved process in all eukaryotes (Hollien, 2013). Hac1, the only characterized component of the UPR pathway in *C. albicans*, is activated after the excision of a 19 bp long intron from its mRNA, in response to tunicamycin‐induced ER stress (Wimalasena et al., 2008). In order to assess the involvement of Ire1 in this *HAC1* mRNA splicing event, we examined the occurrence of unconventional splicing of *HAC1* ^*u*^ (unspliced) mRNA and expression of its target genes following tunicamycin exposure in specific strains of interest. *HAC1* splicing was monitored by (a) performing RT‐PCR using primers across the *HAC1* mRNA intron followed by analysing the PCR products on an agarose gel and (b) using TaqMan probes to minimize background errors to obtain higher efficiency and accuracy during target amplification. The appearance of two RT‐PCR products (unspliced and spliced) in the wild‐type and *ire1* DX comp strain, compared to unstressed cells, where only one product is visible (unspliced), indicated that efficient splicing of the intron occurred in the tunicamycin‐exposed strains (Figure 3a). The absence of a spliced *HAC1* transcript in the *ire1* DX mutant strain suggested that compromised Ire1 expression severely affected the splicing event, demonstrating the requirement of proper Ire1 biosynthesis for efficient splicing of the intron from *HAC1* mRNA. Taqman analysis using specific primer‐probe pairs for the intron (for *HAC1* ^*u*^ pre mRNA) and exon–exon junction (for mature *HAC1* ^*i*^ mRNA) was consistent with the results obtained by RT‐PCR. We observed a 13‐fold induction in spliced *HAC1* ^*i*^ transcript in the wild‐type cells following ER stress, while the induction was compromised (<1.5‐fold) in the *ire1* DX mutant strain (Figure 3b).

Next, to evaluate further the role of Hac1 as the downstream transcription factor of Ire1, we measured the transcript levels of a subset of Hac1‐dependent UPR targets (*SEC61*, *YSY6*, *KAR2*, *orf19.2756* and *ERD2*) known to be upregulated in response to tunicamycin‐induced ER stress (Thomas et al., 2015; Wimalasena et al., 2008). Presuming that the absence of the spliced *HAC1* ^*i*^ transcript should be reflected in the impaired activation of Hac1 targets, we examined the transcript levels of the target genes following tunicamycin exposure (4.7 μM for 2 hr). On treatment with tunicamycin, all of the measured UPR marker genes were significantly upregulated ≥1.5‐fold in wild‐type cells, whereas their expression was compromised in the *ire1* DX mutant strain (≤1.5‐fold induction), correlating well with *HAC1* ^*i*^ transcript levels in these strains (Figure 3c). This observation points to the involvement of the Ire1 protein kinase in activating the UPR in *C. albicans* via splicing of the atypical intron in *HAC1* mRNA. We, therefore, infer that in the *C. albicans* UPR signalling pathway, Hac1 is the transcription factor that functions downstream of Ire1.

To elucidate the contribution of the protein kinase and nuclease functions of Ire1 in the context of ER stress in *C. albicans*, we created strains with impaired kinase or nuclease functions. In *S. cerevisiae*, the kinase and nuclease activities of Ire1 can be segregated by mutating two catalytic residues (D797N and K799N) in the nucleotide binding pocket of the Ire1 kinase (Figure 1a and Figure S1). In *S. cerevisiae*, this mutated Ire1 loses its ability to phosphorylate but retains its RNase activity (Rubio et al., 2011). Likewise, the nuclease active site of *S. cerevisiae* Ire1, present within 10 conserved amino acid residues of its endonuclease domain, contains three conserved residues important for its nuclease activity (Lee et al., 2008). Considering the overall sequence conservation of these residues in *C. albicans* Ire1 (Figure S1), we constructed strains expressing kinase‐dead (*IRE1‐*KD) and nuclease‐dead (*IRE1‐*ND) versions of Ire1. *IRE1‐*KD contains the D890N and K892N mutations in the Ire1 kinase domain, whereas *IRE1‐*ND contains a 10‐amino acid deletion within the Ire1 endonuclease domain (D1161‐Y1170; Figure 1a). The contributions of these domains to ER stress were examined by allowing the constructed mutant strains to grow in the presence of ER stressors (tunicamycin or DTT). The growth defect of the *ire1* DX mutant strain was restored in the strain expressing the wild‐type *IRE1* but not in the strains expressing the *IRE1‐*KD or *IRE1‐*ND mutations in the *ire1* DX mutant strain (Figure 3d). For further validation, both mutants were analysed for their abilities to splice the 19 bp intron from *HAC1* mRNA after tunicamycin exposure. In contrast to the presence of the spliced isoform of *HAC1* ^*i*^ mRNA in the wild‐type and *ire1* DX comp strains, this isoform was absent in all mutated versions of Ire1 under tunicamycin conditions (Figure 3e). Taken together, these data point to the indispensability of the kinase and nuclease domains of Ire1 in dealing with ER stress in *C. albicans*. We conclude that following tunicamycin exposure, Ire1 protein kinase copes with the load of misfolded proteins accumulated in the ER by processing *HAC1* ^*u*^ mRNA in order to activate the UPR and that the Ire1‐Hac1‐mediated UPR pathway is evolutionary conserved in *C. albicans*.

### 2.4 Ire1 supports cell wall and cell membrane homeostasis

**FIGURE 4:**
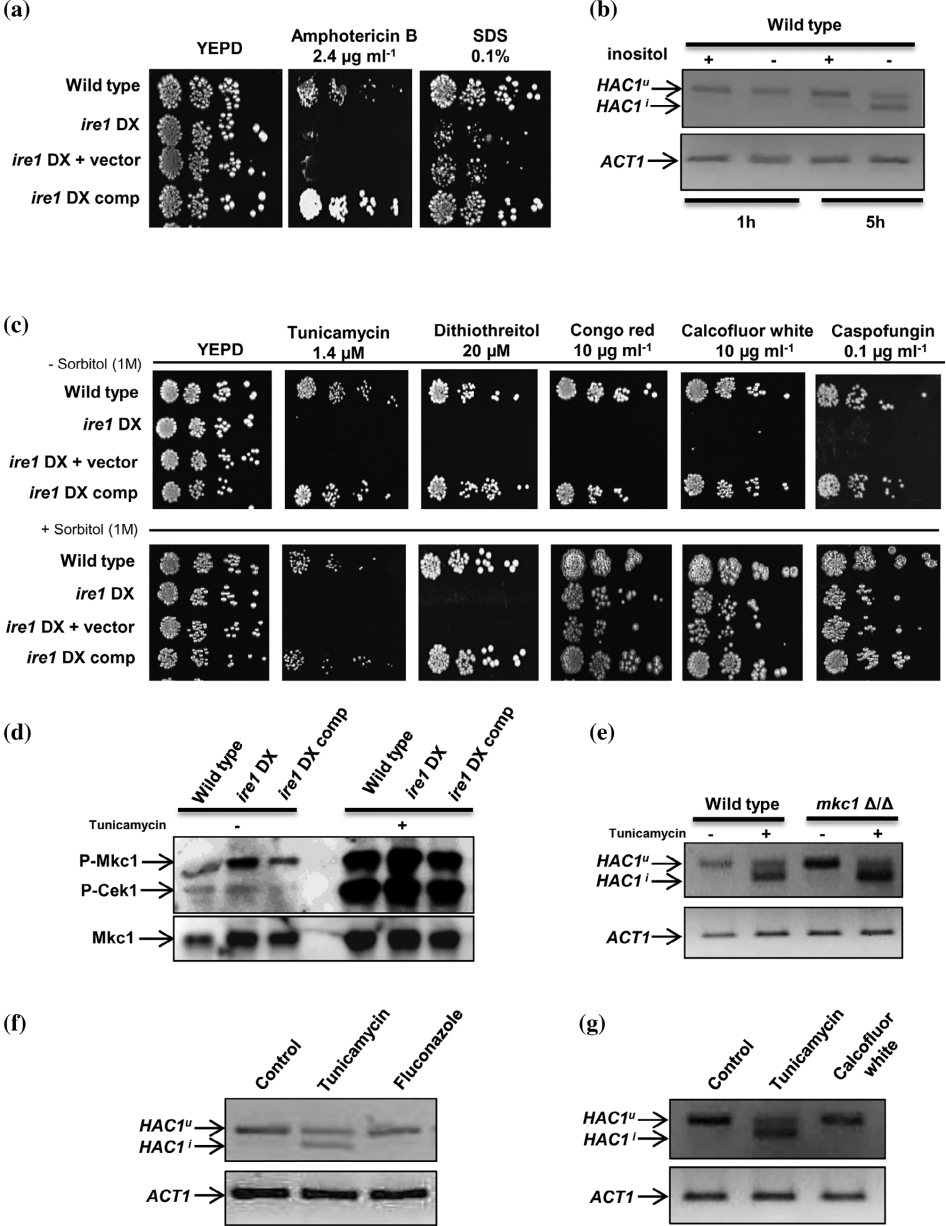
Ire1 impacts cell wall and cell membrane homeostasis in *C. albicans*. (a) Phenotypic comparison of the indicated strains on YEPD plates containing membrane perturbing agents, grown at 30°C for 48 h. (b) RT‐PCR showing *HAC1* splicing (on a 4% agarose gel) in response to inositol depletion over time. cDNA was obtained from the wild type grown in YNB media (with or without inositol). (c) Phenotypic comparison of the indicated strains on different cell wall stressors at indicated concentrations, with or without sorbitol. Plates were incubated at 30°C for 48 hr. (d) Immunoblot showing phosphorylation status of Mkc1 and Cek1 in the indicated strains. Total cell protein was extracted from indicated strains after treatment with 5 μg ml^−1^ tunicamycin for 1 hr. (e) RT‐PCR showing *HAC1* splicing (on a 4% agarose gel) for the wild‐type and *mkc1*Δ/Δ cells exposed to tunicamycin for 1 hr. (f) Analysis of *HAC1* splicing in wild‐type cells in response to cell membrane stress (fluconazole) and (g) cell wall stress (calcofluor white). Tunicamycin‐treated cells are used as a control

*C. neoformans* cells exposed to fluconazole and CFW activate the Ire1‐dependent UPR pathway, suggesting that the UPR serves as the core defence mechanism for cell wall‐ and cell membrane‐induced ER stress. Additionally, deletion of Ire1 and Hac1 results in increased susceptibility to azole antifungal drugs and cell wall stressors in *C. neoformans* and *A. fumigatus* (Cheon et al., 2011; Feng et al., 2011; Jung, Kang, & Bahn, 2013). Since the *ire1* DX mutant strain exhibited increased susceptibility to azole antifungals and cell wall stressors (Figure 1c), we were prompted to evaluate the precise role of Ire1 in regulating susceptibility to these antifungal drugs. As ER is the site for ergosterol and lipid biosynthesis, we presumed that a compromised UPR affecting ER homeostasis and ER‐associated processes could be the underlying basis for the increased susceptibility of the *ire1* DX mutant strain to azole antifungal drugs. In line with this reasoning, our transcriptional profiling data indicated compromised expression of key ergosterol biosynthesis genes (*ERG24*, *ERG11* and *ERG2*) in the *ire1* DX mutant strain that resonated well with the decreased transcript levels of these genes (Figure 2d). We presumed that any perturbation in this pathway could have a direct effect on membrane stability that could be exacerbated by fluconazole‐induced perturbation in sterol homeostasis (Abe, Usui, & Hiraki, 2009). To test this possibility, we examined the response of the *ire1* DX mutant strain to compounds interfering with cell membrane integrity (SDS and amphotericin B). The increased susceptibility of the *ire1* DX mutant strain to these compounds confirmed the requirement of Ire1 in maintaining membrane stability (Figure 4a). Furthermore, based on this observation, we asked if *C. albicans* Ire1 activates the UPR via sensing lipid bilayer stress. Inositol depletion, by inducing membrane stress, can cause ER stress without resulting in the accumulation of misfolded proteins in the ER lumen (Lajoie, Moir, Willis, & Snapp, 2012; Merksamer & Papa, 2010). Consequentially, membrane stress is sensed by both mammalian and *S. cerevisiae* Ire1, resulting in the activation of the UPR (Halbleib et al., 2017; Volmer & Ron, 2015). To test whether *C. albicans* Ire1 responds to membrane stress via *HAC1* splicing, we sought to explore the splicing event in wild‐type cells grown for 1 and 5 hr in growth medium lacking inositol. This experiment shows the occurrence of Ire1‐dependent splicing of *HAC1* mRNA in wild‐type cells grown in medium containing inositol for 5 hr, indicating that growth for long durations in this medium may be inducing ER stress leading to activation of the UPR. In contrast, *HAC1* splicing was more pronounced at 5 hr in medium lacking inositol (Figure 4b), indicating that Ire1 responds to accumulation of misfolded proteins (Figure 3a) as well as to membrane stress by initiating the processing of *HAC1* ^*u*^ mRNA in *C. albicans*, albeit at different time points. Thus, it is likely that Ire1 impacts azole susceptibility via its ability to sense aberrations in membrane homeostasis.

Given the dependency of cell wall biosynthesis on secretory homeostasis, both the UPR and ER quality control mechanisms are essential for maintaining cell wall integrity (Krysan, 2009; Lesage & Bussey, 2006; Levin, 2011; Scrimale, Didone, de Mesy Bentley, & Krysan, 2009). Increased susceptibility of the *ire1* DX mutant strain to cell wall‐damaging agents (Figure 1c) also pointed to the requirement of a healthy working UPR for cell wall maintenance in *C. albicans*. As previous studies have reported that cell wall defects are remediated by the addition of an osmotic stabilizer (e.g., sorbitol) in the growth medium (Chen et al., 2005; Cheon et al., 2011), we analysed our strains for osmoremedial phenotypes in the presence of sorbitol. Inclusion of sorbitol rescued the susceptibility of the *ire1* DX mutant strain to cell wall stressors without affecting susceptibility to the ER stressors, tunicamycin and DTT (Figure 4c). This finding indicates that ER stress‐induced loss of cell viability is not due to an intrinsic cell wall defect in the *ire1* DX mutant strain, and the inability of the mutant to sustain growth in the presence of cell wall stressors is primarily a consequence of impaired secretory homeostasis due to ER stress. Furthermore, compromised expression of Ire1 did not result in changes in cell wall architecture as assessed by transmission electron microscopy and cell wall composition analysis (data not shown). Collectively, these results suggest that defective secretory homeostasis is the basis for increased susceptibility to cell wall stressors in the *ire1* DX mutant strain and implicates Ire1 in maintaining cell wall integrity via its role in maintaining secretory homeostasis in *C. albicans*.

Given that defects in the cell wall activate the Mkc1 MAPK‐regulated cell wall integrity (CWI) pathway, we decided to explore whether defects in the Ire1‐dependent ER stress response pathway can impact the CWI pathway. Hence, we decided to analyse the status of the CWI pathway in wild‐type and *ire1* DX mutant cells in the presence and absence of tunicamycin. The Mkc1 MAPK was constitutively phosphorylated (1.7‐fold) in the *ire1* DX mutant strain, compared to the wild‐type strain (Figure 4d). Tunicamycin treatment resulted in significantly enhanced levels of phosphorylated Mkc1 in the *ire1* DX mutant strain (2.6‐fold), compared to the treated wild‐type strain (Figure 4d). Next, to test if the CWI pathway plays a role in activating the Ire1‐dependent UPR pathway, we monitored the splicing of *HAC1* mRNA in the tunicamycin‐treated *mkc1*Δ/Δ (MAPK regulating CWI pathway) mutant strain (Navarro‐García, Sánchez, Pla, & Nombela, 1995). During tunicamycin treatment, *HAC1* ^*u*^ mRNA was processed in the *mkc1*Δ/Δ cells similar to wild‐type levels (Figure 4e), suggesting that the Mkc1 MAPK pathway does not directly regulate the splicing of *HAC1* mRNA during ER stress. These findings indicate that (a) the absence of Ire1 results in cell wall defects but does not impair the activation of CWI pathway, (b) ER stress activates both the Ire1‐Hac1‐dependent UPR and the CWI pathways and (c) Ire1‐dependent UPR is essential for circumventing cell membrane and cell wall stress in *C. albicans*.

The stress‐responsive calcineurin pathway is involved in circumventing both ER and cell wall stress as the *C. albicans* calcineurin mutant (*cmp1*Δ/Δ) exhibits increased susceptibility to tunicamycin and cell wall damaging agents (Bader, Bodendorfer, Schröppel, & Morschhäuser, 2003; Thomas et al., 2015). In order to examine the effect of the calicneurin pathway in activating the Ire1‐dependent UPR pathway, we analysed the processing of *HAC1* ^*u*^ mRNA in the *cmp1*Δ/Δ cells. We show that the levels of spliced *HAC1* mRNA in tunicamycin‐treated *cmp1*Δ/Δ cells remained similar to the treated wild‐type cells (Figure S4), indicating that, similar to the Mkc1 MAPK pathway, the calcineurin pathway does not directly regulate the processing of the *HAC1* ^*u*^ mRNA in this pathogenic fungus.

Next, we asked whether the role of Ire1 in regulating susceptibility of *C. albicans* to azole antifungal drugs and cell wall stressors is dependent on the activation of the UPR pathway via the unconventional splicing of *HAC1* mRNA. To answer this question, we examined if wild‐type cells treated with fluconazole (10 μg ml^−1^) or CFW (20 μg ml^−1^) can activate *HAC1* mRNA splicing over time (1, 3 and 5 hr) as this event is considered a hallmark for activation of the UPR. *HAC1* ^*u*^ mRNA was not processed at any of the tested time points in fluconazole‐ and CFW‐treated wild‐type cells (Figure 4f,g and Figure S3). This finding indicates that ER stress induced by azole and cell wall stressors is essentially different from that induced by tunicamycin and possibly does not necessitate *HAC1* splicing up to 5 hr. It is possible that, unlike tunicamycin, other stressors do not result in the rapid accumulation of misfolded proteins and hence do not initiate Ire1‐dependent *HAC1* splicing at the time points tested. Overall, these results place Ire1 as the link between protein quality control and membrane homeostasis for the maintainance of cell wall/membrane integrity in *C. albicans*.

### 2.5 Ire1 activity impacts virulence traits of *C. albicans*

**FIGURE 5:**
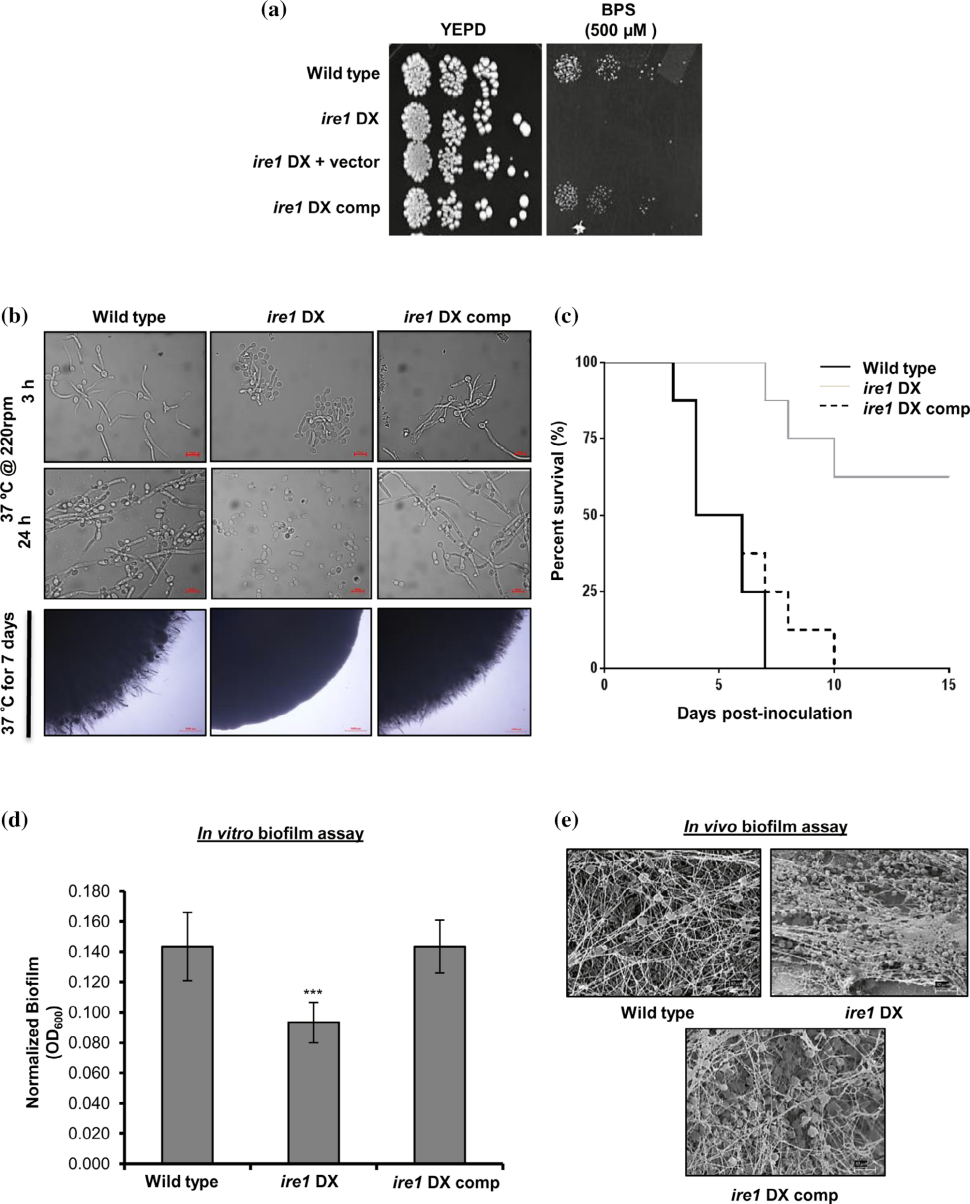
Ire1 regulates pathogenicity traits in *C. albicans*. (a) For phenotypic comparison, indicated strains were spotted on YEPD plates supplemented with BPS. Plates were incubated at 30°C for 48 hr. (b) Indicated strains were grown in filamentation‐inducing Spider medium; in liquid (upper panels) and on solid surface (lower panel) at 37°C and bud‐to‐hyphae transition was monitored over time by microscopy. (c) Kaplan–Meier survival curves showing percent survival of *ire1* DX mutant strain in a mouse model of systemic infection. The mice were infected intravenously with cells (10^6^ cells/mouse) on Day 0 of the experiment, and survival was monitored for 15 days post infection (Log‐rank [Mantel‐Cox] test; *p* = .0004). (d) in vitro biofilm formation for wild type, *ire1* DX, and *ire1* DX comp in Spider medium after 24 hr of culture growth was monitored. OD_600_ readings were measured for adhered biofilms after removal of the medium and normalized to the wild‐type strain (OD_600_ set to 1.0) and the mean ± *SD* is shown. The asterisk indicates a significant difference relative to wild type. A two‐tailed, unpaired *t*‐test was used to determine the statistical relevance. ^***^ *p* < .001. (e) in vivo biofilm formation was assessed by inoculating central venous catheters with the indicated strains, introduced into rats and incubated for 24 hr. Catheters were removed and visualized by scanning electron microscopy (SEM)

The abilities of *C. albicans* to (a) adapt to host microenvironments with different iron content, (b) switch from the yeast to hyphal morphology and (c) form biofilms on mucosal surfaces are important fungal processes for establishing a successful infection in a mammalian host. All of these virulence traits are supported by an intact Ire1‐dependent secretory pathway (Cheon et al., 2011; Feng et al., 2011; Jung et al., 2013). During infection in vivo, *C. albicans* routinely encounters iron deficiency due to iron sequestration by the host (Ramanan, 2000). In order to circumvent iron‐starved environments, *C. albicans* activates the expression of essential iron regulon genes (Chen & Noble, 2012) that facilitate iron acquisition from the host (Fourie, Kuloyo, Mochochoko, Albertyn, & Pohl, 2018). Transcriptional profiling analysis revealed downregulation of genes associated with maintaining iron homeostasis (*FET34*, *RBT5*, *FET3* and *FRE10*), pointing towards the remodelling of iron‐dependent metabolic pathways in the *ire1* DX mutant strain. To this end, we tested the ability of the *ire1* DX mutant strain to grow in iron‐limiting medium that contains the iron chelator bathophenanthroline disulfonate (BPS). The *ire1* DX mutant strain failed to grow on medium containing BPS, while the reconstituted strain restored the growth defect to wild‐type levels (Figure 5a), indicating that functional Ire1 may have a role in facilitating adaptation of *C. albicans* to low iron stress.

Considering the differential regulation of genes involved in hyphal morphogenesis and biofilm formation in the *ire1* DX mutant strain (Figure 2a), we also assessed the contribution of Ire1 in regulating these important virulence traits. The ability of the *ire1* DX mutant strain to form hyphae compared to the wild‐type and complemented strains was assessed in different filament‐inducing medium at 37°C. The *ire1* DX mutant strain exhibited defects in hyphal development in liquid media as well as on solid surfaces (Figure 5b). Hyphal development in the *ire1* DX mutant strain was reduced to 1% compared to 71% in the wild‐type and *ire1* DX comp strains, after 3 hr of growth in Spider medium with a commensurate increase in pseudohyphal cells (66%) and budding yeast cells (33%; Figure 5b). The hyphal defect in the *ire1* DX mutant strain was more pronounced after 24 hr as evident by the presence of a large proportion of yeast cells (Figure 5b). A similar hyphal defect in the *ire1* DX mutant strain was also observed in all media tested (Figure S5), confirming the impact of Ire1 on hyphal morphogenesis in *C. albicans*. These results led us to examine the virulence of the *ire1* DX mutant strain in a mouse model of systemic infection. Even after 15 days post infection, mice infected with the *ire1* DX mutant strain remained healthy and asymptomatic, while mice infected with the wild‐type and *ire1* DX comp strains failed to survive beyond 7 and 10 days post infection, respectively (Figure 5c).

As the ability to adhere to different surfaces for efficient biofilm formation is considered a key virulence trait in *C. albicans*, we tested the strains of interest for their abilities to adhere to and form biofilms on polystyrene plates. The biofilm‐forming abilities of the *ire1* DX mutant strain was reduced by ~1.5‐fold relative to the wild‐type strain (Figure 5d). To assess the ability of the *ire1* DX mutant strain to form biofilms in vivo in a rat central venous catheter biofilm model system, implanted catheters were inoculated with the strains shown in Figure 5e, and biofilm formation was visualized after 24 hr by scanning electron microscopy (SEM) (Andes et al., 2004). While the wild‐type and the *ire1* DX comp strains formed normal and mature biofilms, the *ire1* DX mutant strain was defective in normal biofilm formation, evident from the presence of only yeast cells in the catheter lumen (Figure 5e). These findings demonstrate that Ire1 is required for normal biofilm development both in vitro and in vivo in *C. albicans*. Taken together, these results indicate that several important *C. albicans* virulence traits are intertwined with the ability of Ire1 to support secretory homeostasis in *C. albicans*.

## 3 DISCUSSION

**FIGURE 6:**
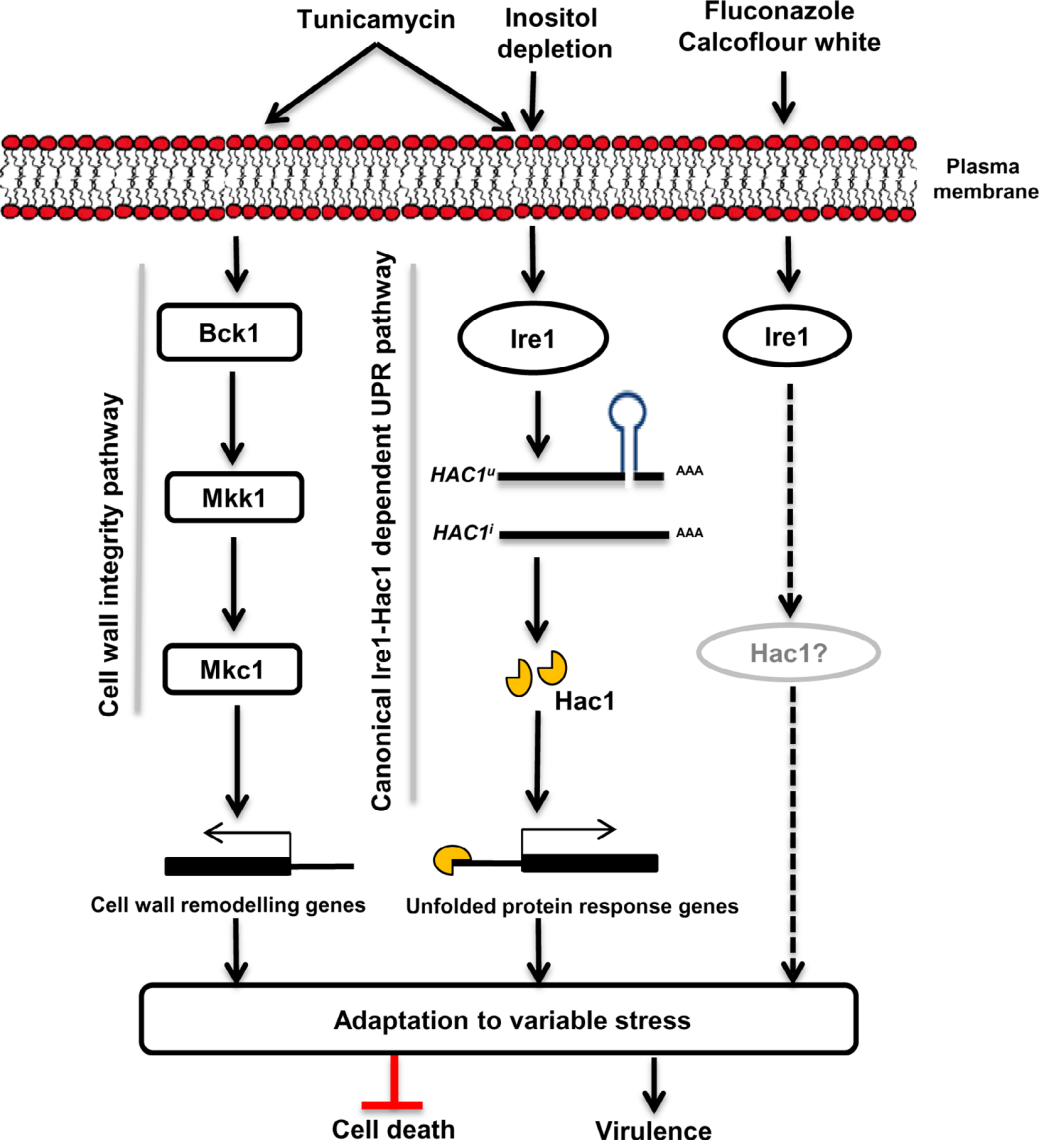
Schematic depicting the involvement of Ire1 in circumventing multiple stresses in *C. albicans*. Accumulation of unfolded proteins in the endoplasmic reticulum (ER) lumen, caused by tunicamycin‐induced ER stress, results in the activation of the canonical unfolded protein response (UPR) branch via Ire1. This protein kinase also responds to membrane stress, generated by depleting the growth medium of inositol, to activate the UPR via processing of *HAC1* ^*u*^ *mRNA*. Ire1 mediates the splicing of pre‐mRNA (*HAC1* ^*u*^) to mature mRNA (*HAC1* ^*i*^) by virtue of its endoribonuclease activity. The spliced *HAC1* synthesizes functional Hac1 protein and activates the UPR target genes for survival during ER stress. Processing of *HAC1* ^*u*^ mRNA is not necessitated in response to other stress stimuli such as cell wall and cell membrane stress, though the involvement of the splicing event at longer time points (beyond 5 hr) cannot be ruled out (indicated by dotted arrows). ER stress also activates complementary signalling pathways such as the cell wall integrity pathway that may operate directly or indirectly with the Ire1‐dependent UPR pathway to coordinate cellular responses in *C. albicans*

The UPR regulated by the highly conserved Ire1‐Hac1 signalling pathway allows human fungal pathogens to counteract host‐mediated ER stress during infection. The splicing of a 19 bp intron in the *C. albicans HAC1* mRNA, initiated during ER stress, is essential for its translation (Wimalasena et al., 2008). While this unconventional splicing event is known to be dependent on Ire1 in most pathogenic fungi, its dependence on Ire1 in *C. albicans* remains unexplored. In this study, we demonstrate the requirement of *C. albicans* Ire1 in circumventing ER stress induced by multiple stressors and its influence on several important virulence traits (Figure 6).

The dependency of Ire1 on Hac1 during ER stress induced by treatment with tunicamycin is evident based on our findings that compromising Ire1 function results in (a) growth defects in the presence of ER stress‐inducing agents (tunicamycin and DTT), (b) abrogated intron splicing from *HAC1* mRNA and (c) decreased expression of Hac1‐dependent ER stress response genes (Figures 1c and 3a–c)). The inability of the *ire1* DX mutant strain to grow on ER stressors and process *HAC1* ^*u*^ mRNA was rescued by intact *IRE1* but not by the *IRE1*‐KD (kinase dead) or *IRE1*‐ND (nuclease dead)‐mutant versions of Ire1, indicating that both the kinase and nuclease activities are indispensible in facilitating the capacity of this protein kinase to counter ER stress (Figure 3d,e). Overall, these results highlight the reliance of *C. albicans* on the canonical Ire1‐Hac1 UPR pathway for ER stress response and homeostasis.

The UPR is required for the removal of misfolded proteins from the ER and is also involved in a broad range of cellular processes related to secretory homeostasis, which can be inferred from our transcriptome analysis (Figure 2a). The impact of Ire1 on various cellular processes was supported by our transcriptome data and by the finding that the *ire1* DX mutant strain exhibited growth defect in the presence of multiple stressors (Figure 1c). Furthermore, the mutant strain also exhibited changes in the expression of genes encoding proteins that promote virulence‐associated processes such as iron assimilation, cell wall biogenesis, hyphal morphogenesis and biofilm formation (Figure 2). Concordantly, the mutant was impaired in these same virulence‐associated processes (Figure 5). We posit that Ire1 regulates *C. albicans* pathogenesis by integrating the expression of virulence‐related traits with ER‐dependent maintenance of secretory homeostasis. Thus, our data support the idea that Ire1 plays a role in mediating multiple cellular processes via its ability to maintain secretory homeostasis in *C. albicans*.

Ire1‐dependent activation of the UPR occurs not only upon the accumulation of misfolded proteins but also during lipid bilayer stress. A link between Ire1, lipid homeostasis and UPR activation has been established in previous studies. In *S. cerevisiae*, inositol depletion is known to cause perturbations in lipid metabolism that results in the activation of Ire1‐dependent UPR activity via an amphipathic helix region present within the transmembrane helix of Ire1 (Halbleib et al., 2017; Promlek et al., 2011). In line with this idea, *C. albicans* Ire1 also responded to membrane abberations, induced by inositol depletion, by promoting the splicing of *HAC1* mRNA (Figure 4b). Considering that inositol depletion in *S. cerevisiae* does not cause protein damage in the ER lumen (Promlek et al., 2011), we speculate that activation of the Ire1‐dependent UPR pathway in *C. albicans* may similarly not be exclusively related to the protein load in the ER. In the absence of inositol, the slow activation (5 hr after stimulus onset vs. 1 hr after tunicamycin treatment) of the UPR could be either due to the presence of residual intracellular inositol or membrane stress caused by inositol depletion that is not considered as severe as tunicamycin‐induced ER stress (Figure 4b), similar to the obervations made with the *S. cerevisiae* Ire1 (Promlek et al., 2011). Thus, our study reveals a link between lipid homeostasis and the Ire1‐dependent UPR pathway in *C. albicans*. Based on this finding, we presumed that fluconazole‐induced perturbations in sterol homeostasis may be sufficient to promote the Ire1‐dependent processing of *HAC1* ^*u*^ mRNA. On the contrary, we observed that fluconazole treatment of the wild‐type cells did not necessitate the processing of *HAC1* ^*u*^ mRNA over time (Figure 4f). Likewise, interfering with cell wall integrity (calcofluor white treatment) that indirectly affects membrane homeostasis (Promlek et al., 2011) also did not necessitate the processing of *HAC1* ^*u*^ mRNA (Figure 4g). Whether *C. albicans* Ire1 responds to these cell wall/membrane stressors in a completely Hac1‐independent manner even at time points that extend beyond 5 hr is a question that requires further experimental exploration. Nevertheless, our data show that Ire1 responds to the accumulation of misfolded proteins as well as membrane stress to activate the UPR in *C. albicans*. As the accumulation of misfolded proteins (resulting from tunicamycin treatment) causes a sudden increase in the demand for secretion and proper protein folding machinery, the cell quickly activates Ire1‐dependent UPR via *HAC1* splicing to allow accelerated recovery of ER homeostasis. On the other hand, stimuli that generate membrane stress do not result in an immediate accumulation of misfolded proteins and hence are slow in activating the UPR and may (as is the case with inositol depletion) or may not (as is the case with fluconazole or calcofluor white treatment) be routed through the processing of *HAC1* ^*u*^ mRNA in *C. albicans*. In the latter situations, it is possible that *C. albicans* may resort to the process of regulated Ire1‐dependent decay (RIDD) for controlling the load of misfolded proteins in the ER lumen. RIDD involves Ire1‐mediated selective degradation of ER‐associated mRNAs that results in a reduction of the protein influx and load of misfolded proteins in the ER (Hollien et al., 2009), a process utilized by *C. glabrata* for ER quality control (Miyazaki et al., 2013). Whether or not *C. albicans* uses RIDD for ER quality control is an area that remains unexplored and merits further investigation.

In conclusion, our data demonstrate that the accumulation of misfolded proteins during tunicamycin‐induced acute ER stress induces the UPR by promoting the splicing of *HAC1* ^*u*^ mRNA in an Ire1‐dependent manner in *C. albicans* (Figure 6). Ire1 also markedly affects cell wall integrity and pathogenesis in *C. albicans*. The ability of Ire1 to jointly respond to both membrane and protein homeostasis enables Ire1 to activate the UPR in response to multiple stressors in *C. albicans*. Coping with ER stress also requires activation of complementary signalling pathways such as the Mkc1 MAPK and calicneurin pathways that operate in parallel with the Ire1‐dependent UPR pathway in a manner that does not influence the processing of *HAC1* ^*u*^ mRNA in *C. albicans* (Figure 4e and Figures S4 and S6). It is possible that the ligand‐binding pockets of the KEN domain present at the Ire1 dimer interface (Wiseman et al., 2010) facilitate integration of Ire1 with other pathways. Our findings reveal a clear relationship between Ire1‐dependent UPR activity and virulence in this pathogenic fungus, a common thread among pathogenic fungi. The conservation of the Ire1‐dependent signalling pathway among pathogenic fungi suggests its potential to be exploited for the design of broad‐spectrum antifungal drugs. Future studies focusing on identifying putative differences between the fungal and mammalian Ire1 are required for identification of fungal‐specific molecules to selectively perturb fungal UPR pathways.

## 4 EXPERIMENTAL PROCEDURES

### 4.1 Strain, reagents and growth conditions

Strains, plasmids and oligonucleotides used in this study are listed in Tables S1–S4. All *C. albicans* strains were maintained on YEPD medium (1% yeast extract, 2% bacto peptone, 2% glucose and 2% agar for solidification). Prototropic transformants were selected on a synthetic medium (2% dextrose, 1.7% Difco yeast nitrogen base with ammonium sulfate and auxotrophic supplements). For nourseothricin‐resistant mutants, 200 μg ml^−1^ of nourseothricin (Werner Bioagents, Jena, Germany) was added to the YEPD. To obtain nourseothricin‐sensitive transformants, strains were grown in YPM (1% yeast extract, 2% peptone and 2% maltose) for 8 hr and plated on YPM plates containing 25 μg ml^−1^ nourseothricin for 48 hr. Except for sodium dodecyl sulfate (SDS; Biobasic, Inc.) and DTT (SRL), all the supplements and chemicals; β‐mercaptoethanol, tunicamycin, fluconazole, ketoconazole, voriconazole, amphotericin B, calcofluor white and congo red were purchased from Sigma‐Aldrich. Caspofungin was obtained from Merck & Co. Inc., NJ.

### 4.2 Strain construction

#### 4.2.1 Gene disruption and complementation of *IRE1*

*ire1* DX (CW906)‐mutant strain was constructed by Woolford et al., 2016. The method used for strain construction is briefly described as follows. A heterozygote deletion was constructed in the DAY286 background by replacing one allele of *IRE1* with the *URA3* marker by homologous recombination, and the 5′ region of the second allele was replaced with a constitutive weak promoter, *PGA5*, to obtain the *ire1* DX (diminished expression)‐mutant strain, a strain with diminished expression of *IRE1* (Woolford et al., 2016). *ire1* DX comp strain was constructed by integrating wild‐type *IRE1* allele in the mutant strain background as previously described (Ganguly & Mitchell, 2012). Briefly, the *IRE1* wild‐type allele was amplified using complementation primers (Table S3) from genomic DNA of SC5314 1984, including 1,200 bp upstream and 300 bp downstream sequences of the *IRE1* ORF. The complementation primers used are approximately 80 bp in length and comprise of an adapter sequence followed by a 45‐mer gene‐specific sequence to direct in vivo recombination into the plasmid pDDB78 (~7.5 kb) (Spreghini, Davis, Subaran, Kim, & Mitchell, 2003). The complementing PCR product was co‐transformed along with EcoRI/NotI digested pDDB78 into the *S. cerevisiae* BJ2698 strain (*his1*) to obtain the complementation plasmid containing *IRE1* called pDDB78‐IRE1WT (~12.3 kb). After amplifying in *Escherichia coli*, pDDB78‐IRE1WT and pDDB78 (vector‐only control) were digested with NruI to direct insertion to the *his1* locus of the *ire1* DX mutant strain to obtain marker matched *HIS1* prototrophic strains; SS1 and SS2, respectively. Successful reconstitution of the gene was confirmed by PCR amplifications performed with primers IRE1CompIRE1DET‐F and IRE1CompHIS1DET‐R (Table S3). Restored gene expression was validated by qPCR using *IRE1* specific primers; qIRE1‐F and qIRE1‐R (Table S4).

#### 4.2.2 Site‐directed mutagenesis

The deletion mutants for kinase dead (*IRE1‐*KD) and nuclease dead domains (*IRE1‐*ND) were constructed using two different mutagenic primer pairs. The plasmid pDDB78‐IRE1WT was used as the template to generate pDDB78‐IRE1KD (kinase dead) and pDDB78‐IRE1ND (nuclease dead) plasmids. The pDDB78‐IRE1KD was generated by mutating two residues in *C. albicans* Ire1; aspartic acid (D2668), and lysine (K2674), to asparagines (D890N and K892N, respectively) using primers IRE1KD‐F(2650) and IRE1 KD‐R(2694). Another deletion plasmid, pDDB78‐IRE1ND was created by introducing a 10 residue (D1161‐Y1170) internal deletion within the nuclease domain of Ire1 using primers, IRE1ND‐F(3456) and IRE1ND‐R(3529) (Table S3). For PCR amplifications, Phusion DNA polymerase (New England Biolabs) was used in a 50 μl reaction mixture containing 10 μl of 5X reaction buffer. All amplifications were done using an Eppendorf Cycler. After an initial denaturation at 95°C for 5 min, the samples were subjected to 25 cycles of denaturation (95°C, 1 min), annealing (61°C, 1 min) and extension (68°C, 9 min). PCRs were completed with another 9 min of extension. Obtained mutated plasmid products were digested with DpnI at 37°C for 1 hr and transformed into *E. coli* for amplification. The plasmid was isolated from all Amp^R^ colonies, and restriction site analysis was performed for verification of the desired mutations in the obtained mutation plasmids. Mutation plasmids were also subjected to Sanger sequencing before final transformation in *C. albicans*. Once confirmed for the mutation, the plasmid was linearized with Nru1 and transformed into *ire1* DX background for *HIS1*‐targeted integration to obtain strains expressing kinase dead (SS3) or nuclease dead *IRE1* isoforms (SS4).

### 4.3 Drug susceptibility assays

All strains of interest were grown on YEPD plates overnight. The cells were resuspended in 0.9% saline to an OD_600_ of 0.1. Five microlitre of four serial dilutions (5 × 10^3^ to 5 × 10^5^ cells) of each strain were spotted on to YEPD plates in presence and absence of various stressors. Plates were incubated at 30°C, and growth differences were recorded after 48 hr.

### 4.4 Quantitative real‐time PCR

*C. albicans* strains were grown overnight in YEPD, subcultured from a starting OD_600_ of 0.3 in fresh YEPD and incubated at 30°C till OD_600_ reached 1.0. The desired drugs/compounds of interest were added to the media, and culture was allowed to grow for 1 hr, except for the qPCR for UPR targets where strains were treated for 2 hr. Cells were harvested by centrifugation at 4,000 rpm for 5 min at 4°C from treated and untreated control samples, and total RNA was isolated using the RNeasymini kit (Qiagen). Extracted RNA was treated with DN*ase* I (Thermo Scientific) to remove contaminating DNA, and cDNA was synthesised with a RevertAid H Minus First Strand cDNA synthesis kit (Thermo Scientific) following the manufacturer's protocol. All real‐time PCR reactions were performed in a volume of 25 μl using Thermo Scientific Maxima SYBR Green mix in a 96‐well plate. For relative quantification of gene expression, the comparative C_T_ method was used, where the fold change was determined as 2^−ΔΔC^_T_ (Schmittgen & Livak, 2008). The qPCR primers used in this study were designed by Primer Express 3.0 and are listed in Table S4.

### 4.5 Determination of *HAC1* mRNA splicing

*C. albicans* cells were treated with tunicamycin for 1 hr, and total RNA was prepared by following the procedure described above. cDNA was prepared by using RevertAid H Minus First Strand cDNA synthesis kit (Thermo Scientific), and *HAC1* mRNA splicing was measured by the following:

1. Reverse transcriptase‐PCR (RT‐PCR) using *HAC1* gene‐specific primers HAC1SP (F) and HAC1SP (R), respectively (Table S4), and the PCR product was analysed on 4% agarose gel. *ACT1* was as an internal control.
2. qPCR was performed with TaqMan probe specific for spliced *HAC1* (*HAC1* ^*i*^) or primers recognizing the unspliced *HAC1* (*HAC1* ^*u*^) isoform. TaqMan Universal PCR Master Mix and primer‐probe mixes were obtained from Applied Biosystems by Life Technologies. *ACT1* was used as the internal control, and transcript level of the gene of interest was normalized to *ACT1* levels. Fold changes are means ± *SD* and are derived from three independent RNA preparations.

### 4.6 Transcriptional profiling

(a) For RNA isolation, wild‐type and mutant strains were grown overnight in 10 ml of YEPD at 30°C and 200 rpm, subcultured to an OD_600_ of 0.3 in 10 ml of YEPD medium and grown till mid‐log phase at 30°C and 200 rpm. RNA was extracted from three biological replicates of both strains using an RNeasyminikit (Qiagen). After testing the integrity of these RNA samples by Bioanalyzer (Agilent 2100), microarray experiment was performed. (b) For hybridization, the samples for gene expression were labelled using Agilent Quick‐Amp labeling Kit (p/n5190‐0442). A total of 500 ng each of total RNA was reverse transcribed at 40° C using oligo dT primer tagged to a T7 polymerase promoter and converted to double‐stranded cDNA. Synthesized double‐stranded cDNA was used as a template for cRNA generation. cRNA was generated by in vitro transcription, and the dye Cy3 CTP (Agilent) was incorporated during this step. The cDNA synthesis and in vitro transcription steps were carried out at 40°C. Labelled cRNA was cleaned up using QiagenRNeasy columns (Qiagen, Cat No: 74106) and quality assessed for yields and specific activity using the Nanodrop ND‐1000. The labelled cRNA sample was fragmented at 60°C and hybridized on to a genotypic designed *C albicans*_GXP_8X15k (AMADID No: 26377) arrays. Fragmentation of labelled cRNA and hybridization was done using the gene expression hybridization kit (Agilent Technologies, In situ Hybridization kit, Part Number 5190–0404). Hybridization was carried out in Agilent's Surehyb Chambers at 65°C for 16 hr. The hybridized slides were washed using Agilent Gene Expression wash buffers (Agilent Technologies, Part Number 5188‐5327) and scanned using the Agilent Microarray Scanner (Agilent Technologies, Part Number G2600D). (c) Raw data extraction from images and subsequent analysis were performed using Agilent Feature Extraction software and GeneSpring GX Software V 13.0, respectively. Normalization of the data was done in GeneSpring GX using the 75th percentile shift method, and fold change values were obtained by comparing mutant samples with respect to specific wild‐type samples. Conditional‐based hierarchical clustering was performed to understand the biological variation within replicate samples. Significant differentially up and down regulated genes with *p*‐value .05 in the mutant with respect to wild type were identified. Statistical student *t* test used to calculate *p*‐value among the replicates was calculated based on volcano Plot Algorithm. Heat maps for the differentially regulated genes were generated using Genespring GX Software. Gene ontology (GO) analysis was carried out using the GO Term Finder at the CGD (Candida Genome Database; http://www.candidagenome.org/cgi-bin/GO/goTerm Finder). Upregulated and downregulated genes were analysed separately. The microarray data can be accessed under GEO accession number GSE137822.

### 4.7 Protein extraction and immunoblot analysis

For the Mkc1p phosphorylation blot, overnight cultures were diluted in fresh YEPD media to an OD_600_ of 0.3 and grown until they reached an OD_600_ of 1 at 37°C and 200 rpm. Samples were treated with 5 μg ml^−1^ tunicamycin for 2 hr before they were recovered. The procedures used for cell collection, lysis, protein extraction, fractionation by SDS‐PAGE and transfer to nitrocellulose membranes have been previously described (Martín, Arroyo, Sánchez, Molina, & Nombela, 1993). Anti‐phospho‐p44/42 MAP kinase (Thr202/Tyr204) antibody (Anti‐p42‐44‐P) (Cell Signalling Technology, Inc.) was used to detect dually phosphorylated Mkc1 and Cek1 MAPKs, and polyclonal anti‐Mkc1 antibodies were used for Mkc1 detection (Federico Navarro‐García, Eisman, Fiuza, Nombela, & Pla, 2005). Blot imaging was done by using an Odyssey fluorescence imager (LI‐COR) and quantified using Image Studio Lite (LI‐COR).

### 4.8 Morphogenesis assays

For filamentation in liquid media, cells from an overnight culture grown in YEPD were used to subculture from a starting OD_600_ of 0.3 in fresh filamentation‐inducing media (Spider, Lee and Serum) and incubated for 4–5 hr at 37°C with continuous shaking. Aliquots of the cells were taken out at 1 hr interval for 24 hr, washed with 1X PBS and observed under light microscope for changes in filamentation patterns. For filamentation on solid media, cells from an overnight culture grown in YEPD were washed and approximately 50–100 cells of each strain were plated on YEPD +10% serum, Lee and Spider agar plates and incubated at 37°C for 5 days. Colonies were observed after 5 days for filamentation changes. The plates were photographed by using a Zeiss microscope equipped with a digital camera to record filamentation patternd in different strains.

### 4.9 Virulence assays

Virulence assays were performed following the procedures described previously (Diez‐Orejas et al., 1997; Román, Alonso‐Monge, Miranda, & Pla, 2015). Briefly, *C. albicans* strains SC5314, *ire1* DX and *ire1* DX comp were cultivated overnight in YEPD. Cells were harvested by low‐speed centrifugation followed by washing twice with phosphate‐buffered saline (PBS). 10^6^ yeast cells (in 250 μL PBS) of each strain were inoculated into the lateral tail vein of BALB/c mice, and survival was monitored for 15 days. Postmortem analyses were done with eight animals, and clearance of infection was assessed by CFU counting as described previously (Diez‐Orejas et al., 1997).

### 4.10 Biofilm assays

#### 4.10.1 In vitro biofilm model

In vitro biofilm assays were carried out in Spider medium by growing the biofilm directly on the bottom of the 96‐well polystyrene plates, as described previously (Fox et al., 2015; Lohse et al., 2017). Briefly, strains were grown overnight in YEPD at 30°C for 12–14 hr and diluted to an optical density at OD_600_ of 0.5 in Spider medium. The inoculated plate was covered with a breathable film and incubated at 37°C for 90 min at 250 rpm agitation on an ELMI incubator (ELMI, Ltd. Riga, Latvia) for initial adhesion of cells. Post adhesion, the cells were washed with 200 μl of 1X PBS, and 200 μl of fresh Spider medium was added. The plate was covered with a fresh breathable film and incubated at 37°C for an additional 24 hr at 250 rpm agitation to allow biofilm formation. Following incubation, the film and medium were removed, and the OD_600_ was measured using a standard plate reader to determine the extent of biofilm formation. A well‐containing medium alone was included as contamination control. Statistical significance (*p*‐values) was calculated using a Student's one‐tailed paired *t* test. *p*‐values are as follows: *** <.001.

#### 4.10.2 In vivo *C. albicans* venous catheter biofilm model

A jugular vein rat central venous catheter infection model was used for in vivo biofilm studies, as previously described (Andes et al., 2004). After 24 hr of *C. albicans* infection, catheters were removed from the rat. The distal 2 cm of catheter material was subjected to SEM for assaying biofilm growth.

## CONFLICT OF INTEREST

Clarissa J. Nobile is a cofounder of BioSynesis, Inc., a company developing diagnostics and therapeutics for biofilm formation. The remaining authors declare no conflict of interest.

## AUTHOR CONTRIBUTIONS

**Sneh Lata Panwar, Shabnam Sircaik:** Conceptualization; **Sneh Lata Panwar, Shabnam Sircaik:** Formal analysis; **Sneh Lata Panwar:** Funding acquisition; **Shabnam Sircaik, Elvira Román, Priyanka Bapat, Keunsook K. Lee, David R. Andes:** Investigation; **Sneh Lata Panwar, Jesús Pla, Clarissa J. Nobile, Neil A. R. Gow, David R. Andes:** Resources; **Sneh Lata Panwar, Shabnam Sircaik:** Validation; **Sneh Lata Panwar, Shabnam Sircaik, Keunsook K. Lee, David R. Andes:** Visualization; **Sneh Lata Panwar:** Writing original draft; **Sneh Lata Panwar, Shabnam Sircaik, Jesús Pla, Clarissa J. Nobile:** Writing‐review and editing.

## ETHICS STATEMENT

For virulence assays, animal procedures were performed by following the regulations in the ‘Real Decreto1201/2005, BOE 252’ for the Care and Use of Laboratory Animals of the ‘Ministerio de la Presidencia’, Spain. The protocol used was approved by the Animal Experimentation Committee of the University Complutense of Madrid (Permit Number: BIO2012‐31839‐1). For in vivo biofilm assays, animal procedures were approved by the Institutional Animal Care and Use Committee at the University of Wisconsin according to the guidelines of the Animal Welfare Act, The Institute of Laboratory Animal Resources Guide for the Care and Use of Laboratory Animals and Public Health Service Policy under protocol MV1947.

## Supporting information


**Figure S1** Sequence alignment of the kinase and nuclease domains of Ire1 fungal orthologues. Alignment of Ire1 protein sequences showing homology between different fungal Ire1 orthologues (Clustal Omega and BOXSHADE). The asterisk indicates the conserved catalytic residues (D797 and K799 in *S. cerevisiae*; D890 and K892 in *C. albicans*) in the nucleotide‐binding pocket of Ire1 kinase. The predicted nuclease domain is underlined. The box indicates 10 amino acid residues with the three highly conserved amino acid residues (arrowheads) in the active site of the endonuclease domain.
**Figure S2.** Analysis of *IRE1* expression. (A) RT‐PCR for *IRE1* in wild type, *ire1* DX and *ire1* DX comp (*ire1* DX+ *IRE1*) strains. cDNA was obtained after growing the cells to mid‐log phase at 30° C. PCR amplification was done using *IRE1* gene‐specific primers and product was analysed on a 1% agarose gel. (B) qPCR showing *IRE1* expression in response to different stressors. cDNA was obtained from the wild‐type strains after treatment with ER stressor (4.7 μM tunicamycin), cell wall stressor (20 μg ml^−1^ calcoflour white), cell membrane stressors (10 μg ml^−1^ fluconazole) and iron depletion (50 μM BPS) for 1 hr. Fold change is calculated by 2^−ΔΔC^_T_, normalized to *ACT1* (endogenous control). Values are mean ± SD derived from three independent RNA preparations.
**Figure S3.** RT‐PCR showing *HAC1* splicing in response to different stressors. cDNA was obtained from wild‐type *C. albicans* after treatment with cell wall stressor (20 μg ml^−1^ calcoflour white) and cell membrane stressor (10 μg ml^−1^ fluconazole) over time. PCR amplification was done using primers flanking the *HAC1* intron, and product was analysed on a 4% agarose gel. The size difference between *HAC1* (*HAC1* ^*i*^) or unspliced *HAC1* isoform (*HAC1* ^*u*^) form of cDNA is 19 bp.
**Figure S4**. RT‐PCR showing *HAC1* splicing in calcineurin mutant. cDNA was obtained from indicated strain after treatment with 4.7 μM tunicamycin for 1 hr. PCR amplification was done using primers flanking the *HAC1* intron, and product was analysed on a 4% agarose gel. The size difference between *HAC1* (*HAC1* ^*i*^) and unspliced *HAC1* isoform (*HAC1* ^*u*^) form of cDNA is 19 bp.
**Figure S5.** Ire1‐dependent UPR is required for hyphal morphogenesis. Indicated strains were grown in filamentation‐inducing media at 37°C, and bud‐to‐hyphae transition was monitored over time by microscopy (A) in liquid and (B) on solid surface.

**Table S1** Supporting information

## Data Availability

The data that support the findings of this study are openly available in Gene Expression Omnibus at www.ncbi.nlm.nih.gov/geo, Accession number GSE137822.
