# Supplementary material for: The protein kinase Ire1 impacts pathogenicity of Candida albicans by regulating homeostatic adaptation to endoplasmic reticulum stress
Source: Cell Microbiol. 2021 Jan 26;23(5):e13307. doi: 10.1111/cmi.13307 (PMC8044019; doi:10.1111/cmi.13307)
Supplement: Supplementary file 1 — Figure S1 Sequence alignment of the kinase and nuclease domains of Ire1 fungal orthologues. Alignment of Ire1 protein sequences showing homology between different fungal Ire1 orthologues (Clustal Omega and BOXSHADE). The asterisk indicates the conserved catalytic residues (D797 and K799 in S. cerevisiae; D890 and K892 in C. albicans) in the nucleotide‐binding pocket of Ire1 kinase. The predicted nuclease domain is underlined. The box indicates 10 amino acid residues with the three highly conserved amino acid residues (arrowheads) in the active site of the endonuclease domain. Figure S2. Analysis of IRE1 expression. (A) RT‐PCR for IRE1 in wild type, ire1 DX and ire1 DX comp (ire1 DX+ IRE1) strains. cDNA was obtained after growing the cells to mid‐log phase at 30° C. PCR amplification was done using IRE1 gene‐specific primers and product was analysed on a 1% agarose gel. (B) qPCR showing IRE1 expression in response to different stressors. cDNA was obtained from the wild‐type strains after treatment with ER stressor (4.7 μM tunicamycin), cell wall stressor (20 μg ml−1 calcoflour white), cell membrane stressors (10 μg ml−1 fluconazole) and iron depletion (50 μM BPS) for 1 hr. Fold change is calculated by 2−ΔΔC T, normalized to ACT1 (endogenous control). Values are mean ± SD derived from three independent RNA preparations. Figure S3. RT‐PCR showing HAC1 splicing in response to different stressors. cDNA was obtained from wild‐type C. albicans after treatment with cell wall stressor (20 μg ml−1 calcoflour white) and cell membrane stressor (10 μg ml−1 fluconazole) over time. PCR amplification was done using primers flanking the HAC1 intron, and product was analysed on a 4% agarose gel. The size difference between HAC1 (HAC1 i) or unspliced HAC1 isoform (HAC1 u) form of cDNA is 19 bp. Figure S4. RT‐PCR showing HAC1 splicing in calcineurin mutant. cDNA was obtained from indicated strain after treatment with 4.7 μM tunicamycin for 1 hr. PCR amplification was done using primers [file CMI-23-e13307-s002.pdf]

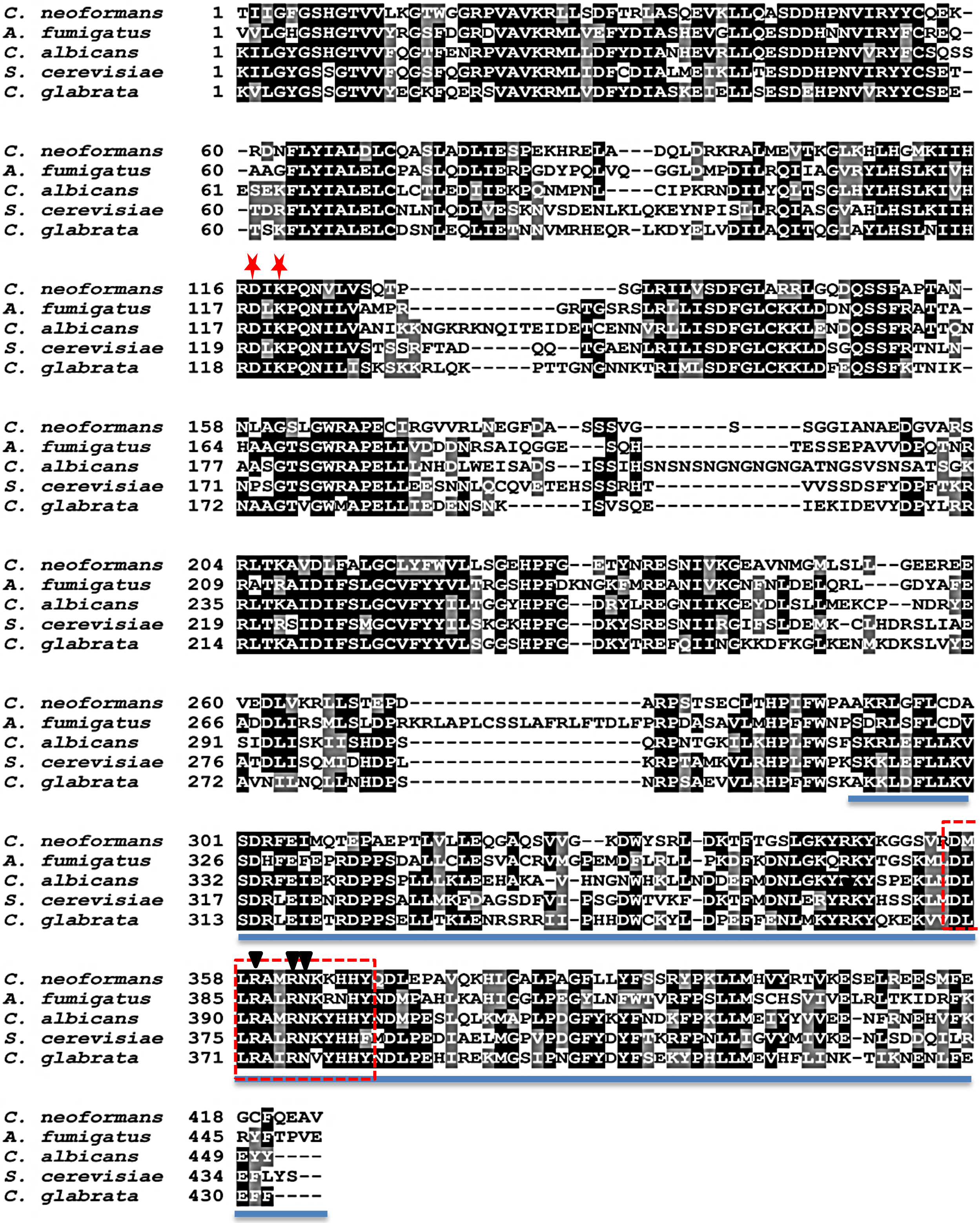
C. neoformans 1 TIIGFGSHGTVVLKGTWGGRPVAVKRLLSDFTRLASQEVKLLQASDDHPNVIRYYCQEK-
A. fumigatus 1 VVLGHGSHGTVVYRGSFDGRDVAVKRMLVEFYDIASHEVGLLQESDDHNNVIRYFCREQ-
C. albicans 1 KILGYGSHGTVVFQGTFENRPVAVKRMLLDFYDIANHEVRLLQESDDHPNVVRYFCSQSS
S. cerevisiae 1 KILGYGSSGTVVFQGSFQGRPVAVKRMLIDFCDIALMEIKLLTESDDHPNVIRYYCSET-
C. glabrata 1 KVLGYGSSGTVVYEGKFQERSVAVKRMLVDFYDIASKEIELLSESDEHPNVVRYYCSEE-
C. neoformans 60 -RDNFLYIALDLCQASLADLIESPEKHRELA---DQLDRKRALMEVTKGLKHLHGMKIIH
A. fumigatus 60 -AAGFLYIALELCPASLQDLIERPGDYPQLVQ--GGLDMPDILRQIIAGVRYLHSLKIVH
C. albicans 61 ESEKFLYIALELCLCTLEDIIEKPQNMPNL----CIPKRNDILYQLTSGLHYLHSLKIVH
S. cerevisiae 60 -TDRFLYIALELCNLNLQDLVESKNVSDENLKLQKEYNPISLLRQIASGVAHLHSLKIIH
C. glabrata 60 -TSKFLYIALELCDSNLEQLIETNNVMRHEQR-LKDYELVDILAQITQGIAYLHSLNIIH
C. neoformans 116 RDIKPQNVLVSQTP------------------SGLRILVSDFGLARRLGQDQSSFAPTAN-
A. fumigatus 117 RDLKPQNILVAMPR-------------GRTGSRSLRLLISDFGLCKKLDDNQSSFRATTA-
C. albicans 117 RDIKPQNILVANIKKNGKRKNQITEIDETCENNVRLLISDFGLCKKLENDQSSFRATTQN
S. cerevisiae 119 RDLKPQNILVSTSSRFTAD------QQ--TGAENLRILISDFGLCKKLDSGQSSFRTNLN-
C. glabrata 118 RDIKPQNILISKSKKRLQK-----PTTGNGNNKTRIMLSDFGLCKKLDFEQSSFKTNIK-
C. neoformans 158 NLAGSLGWRAPECIRGVVRLNEGFDA--SSSVG-------S-----SGGIANAEDGVARS
A. fumigatus 164 HAAGTSGWRAPELLVDDDNRSAIQGGE---SQH------------TESSEPAVVDPQTNR
C. albicans 177 AASGTSGWRAPELLLNHDLWEISADS--ISSIHSNSNSNGNGNGNGATNGSVSNSATSGK
S. cerevisiae 171 NPSGTSGWRAPELLEESNNLQCQVETEHSSSRHT------------VVSSDSFYDPFTKR
C. glabrata 172 NAAGTVGWMAPELLIEDENSNK------ISVSQE------------IEKIDEVYDPYLRR
C. neoformans 204 RLTKAVDLFALGCLYFWVLLSGEHPFG--ETYNRESNIVKGEAVNMGMLSLI--GEEREE
A. fumigatus 209 RATRAIDIFSLGCVFYYVLTRGSHPFDKNGKFMREANIVKGNFNLDELQRL---GDYAFE
C. albicans 235 RLTKAIDIFSLGCVFYYILTGGYHPFG--DRYLREGNIIKGEYDLSLLMEKCP--NDRYE
S. cerevisiae 219 RLTRSIDIFSMGCVFYYILSKGKHPFG--DKYSRESNIIRGIFSLDEMK-CLHDRSLIAE
C. glabrata 214 RLTKAIDIFSLGCVFYYVLSGGSHPFG--DKYTREFQIINGKKDFKGLKENMKDKSLVYE
C. neoformans 260 VEDLVKRLLSTEPD--------------------ARPSTSECLTHPIFWPAAKRLGFLCDA
A. fumigatus 266 ADDLIRSMLSLDPRKRLAPLCSSLAFRLFTDLFPRPDASAVLMHPFFWNPSDRLSFLCDV
C. albicans 291 SIDLISKIISHDPS--------------------QRPNTGKILKHPLFWSFSKRLEFLLKV
S. cerevisiae 276 ATDLISQMIDHDPL--------------------KRPTAMKVLRHPLFWPKSKKLEFLLKV
C. glabrata 272 AVNIILNQLLNHDPS-------------------NRPSAEVVLRHPFFWSKAKKLDFLLKV
C. neoformans 301 SDRFEIMQTEPAEPTLVLLEQGAQSVVG--KDWYSRL-DKTFTGSLGKYRKYKGGSVRDM
A. fumigatus 326 SDHFEFEPRDPPSDALLCLESVACRVMGPEMDFLRLL-PKDFKDNLGKQRKYTGSKMLDL
C. albicans 332 SDRFEIEKRDPPSPLLLKLEEHAKA-V-HNGNWHKLLNDDEFMDNLGKYRKYSPEKLNDL
S. cerevisiae 317 SDRLEIENRDPPSALLMKFDAGSDFVI-PSGDWTVKF-DKTFMDNLERYRKYHSSKLMDL
C. glabrata 313 SDRLEIETRDPPSELLTKLENRSRRII-PHHDWCKYL-DPEFFENLMKYRKYQKEKVIDL
C. neoformans 358 LRAMRNKKHHYQDLEPAVQKHLGALPAGFLLYFSSRYPKLLMHVYRTVKESELREESMFE
A. fumigatus 385 LRALRNKRNHYNDMPAHLKAHIGGLPEGYLNFWTVRFPSLLMSCHSVIVELRLTKIDRFK
C. albicans 390 LRAMRNKYHHYNDMPESLQLKMAPLPDGFYKYFNDKFPKLLMEIYYVVEE-NFRNEHVFK
S. cerevisiae 375 LRALRNKYHHFMDLPEDIAELMGPVPDGFYDYFTKRFPNLLIGVYMIVKE-NLSDDQILR
C. glabrata 371 LRAIRNVYHHYNDLPEHIREKMGSIPNGFYDYFSEKYPHLLMEVHFLINK-TIKNENLFE
C. neoformans 418 GCFQEAV
A. fumigatus 445 RYFTPVE
C. albicans 449 EYY----
S. cerevisiae 434 EFLYS--
C. glabrata 430 EFF----

(A)

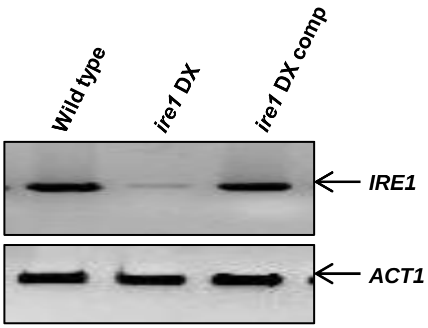
Wild type
ire1 DX
ire1 DX comp
IRE1
ACT1

(B)

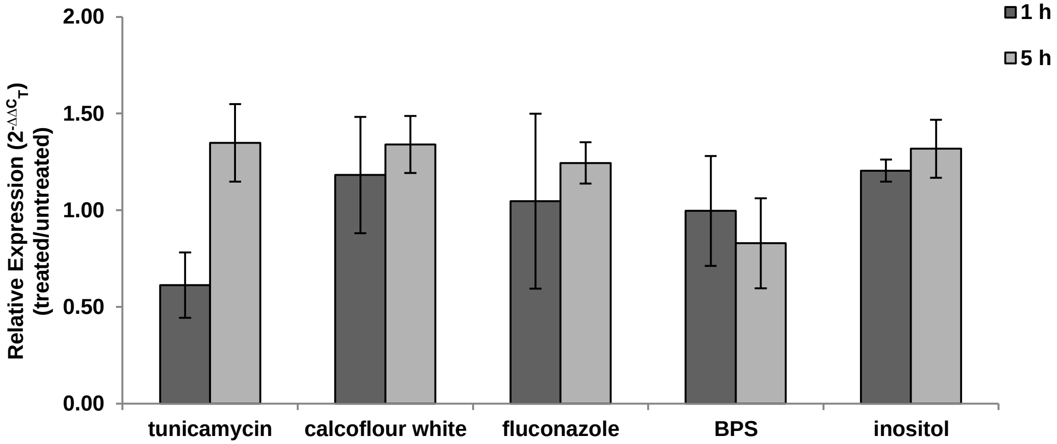
1 h
5 h
Relative Expression (2-ΔΔCT)
(treated/untreated)
2.00
1.50
1.00
0.50
0.00
tunicamycin
calcoflour white
fluconazole
BPS
inositol

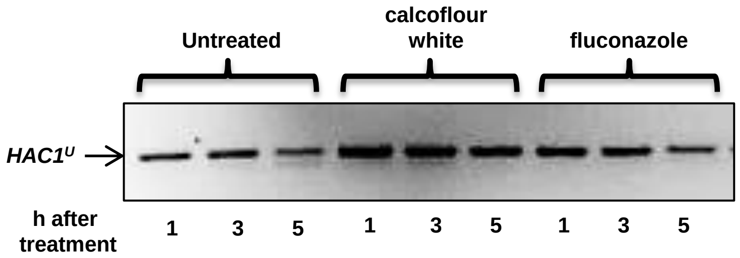

Untreated
calcoflour white
fluconazole
HAC1U
h after treatment
1
3
5
1
3
5
1
3
5

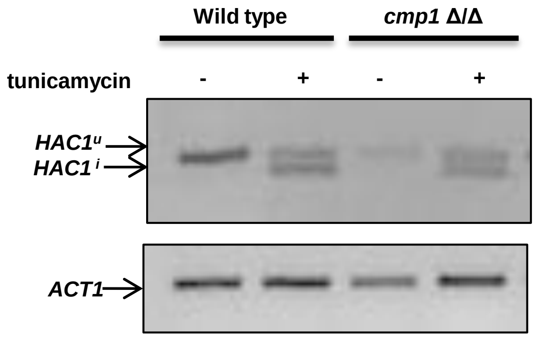
Wild type
cmp1 Δ/Δ
tunicamycin
-
+
-
+
HAC1u
HAC1 i
ACT1

(A)

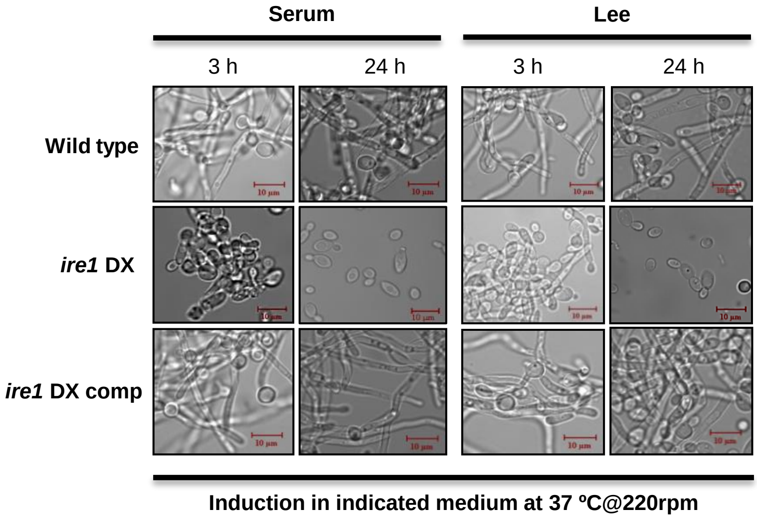
Serum
Lee
3 h
24 h
3 h
24 h
Wild type
ire1 DX
ire1 DX comp
Induction in indicated medium at 37 ºC@220rpm

(B)

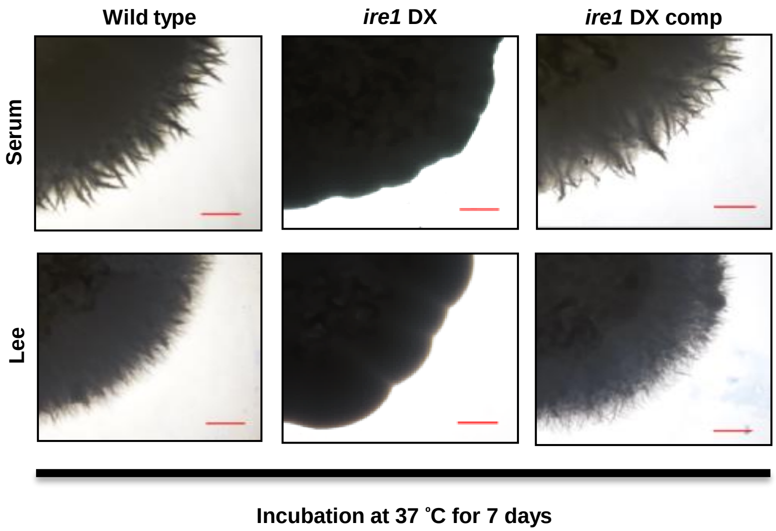
Wild type
ire1 DX
ire1 DX comp
Serum
Lee
Incubation at 37 ˚C for 7 days
