# Supplementary material for: The protein kinase Ire1 impacts pathogenicity of Candida albicans by regulating homeostatic adaptation to endoplasmic reticulum stress
Source: Cell Microbiol. 2021 Jan 26;23(5):e13307. doi: 10.1111/cmi.13307 (PMC8044019; doi:10.1111/cmi.13307)
Supplement: Supplementary file 2 — Table S1 Supporting information [file CMI-23-e13307-s001.pdf]

Table S1: Strains used in this study.

Strain	Parent	Genotype	Source of reference
**SC5314**	Wild type (*C. albicans*)		(Gillum et al., 1984)
**DAY286**	Wild type (*C. albicans*)	*ura3Δ iro1Δ::λimm434 ARG4:URA3:arg4::hisG his1::hisG*<br>*ura3Δ iro1Δ::λimm434 arg4::hisG his1::hisG*	(Woolford et al., 2016)
**CW906**	DAY286	*ura3Δ iro1Δ::λimm434 arg4::hisG his1::hisG ARG4:IRE1-DX1*<br>*ura3Δ iro1Δ::λimm434 arg4::hisG his1::hisG Δire1::URA3*	(Woolford et al., 2016)
**SS1**	*ire1* DX	*ura3Δ iro1Δ::λimm434 arg4::hisG his1::hisG::pHIS1::IRE1 ARG4:IRE1-DX1*<br>*ura3Δ iro1Δ::λimm434 arg4::hisG his1::hisG Δire1::URA3*	This study
**SS2**	*ire1* DX	*ura3Δ iro1Δ::λimm434 arg4::hisG his1::hisG::pHIS1 ARG4:IRE1-DX1*<br>*ura3Δ iro1Δ::λimm434 arg4::hisG his1::hisG Δire1::URA3*	This study
**SS3**	*ire1* DX	*ura3Δ iro1Δ::λimm434 arg4::hisG his1::hisG::pHIS1::IRE1*-KD *ARG4:IRE1-DX1*<br>*ura3Δ iro1Δ::λimm434 arg4::hisG his1::hisG Δire1::URA3*	This study
**SS4**	*ire1* DX	*ura3Δ iro1Δ::λimm434 arg4::hisG his1::hisG::pHIS1::IRE1*-ND *ARG4:IRE1-DX1*<br>*ura3Δ iro1Δ::λimm434 arg4::hisG his1::hisG Δire1::URA3*	This study
**CM-1613**	SC5314	*mkc1D::hisG/mkc1D::hisG ura3D::imm434/ura3D::imm434*	(Navarro-García et al., 1995)
**CMP1M3**	CMP1M2	*cmp1::URA3-FLIP/cmp1::FRT*	(Bader et al., 2003)

Table S2: Plasmids used in this study.

Plasmids	Description	Reference
**pDDB78**	Plasmid carrying *HIS1* marker	(Spreghini et al., 2003)
**pDDB78-IRE1WT**	Plasmid carrying *IRE1* wild type gene	This study
**pDDB78-IRE1KD**	Plasmid carrying *IRE1* kinase inactive gene	This study
**pDDB78-IRE1ND**	Plasmid carrying *IRE1* nuclease inactive gene	This study

Table S3: Oligonucleotides used in this study.

Oligonucleotides	Description	Sequence (5′→3′)
**IRE1Comp-F(1200)**	Forward primer for cloning *IRE1* *(NCR+ORF*) in pDDB78	5'-CAATTTCACACAGGAAACAGCTATGACCATGATTACGCCAAGCTCAAATACGAAAGAAGAATAATACATAGAGATTGGTCTTGACTTGA-3'
**IRE1Comp-R(3945)**	Forward primer for cloning *IRE1* *(NCR+ORF)* in pDDB78	5'-GTCGACCATATGGGAGAGCTCCCAACGCGTTGGATGCATAGAACGTCAAGGAATCTAAAATAACTAAACCAAAATCAAATAAAAAAT-3'
**IRE1Comp IRE1DET-F(3101)**	Forward primer for *IRE1* complementation screening (*IRE1* specific)	5’-AAAAGAGGTAGTCGTG-3’
**IRE1Comp HIS1DET-R(9500)**	Reverse primer for *IRE1* complementation screening (*HIS1* specific)	5'-GCTGATGCTATTGTCGATTTG-3'
**IRE1KD-F(2650)**	Forward primer for *IRE1* kinase domain site directed mutation	5'-TTAAAAATTGTGCATCGTAATATTAATCCACAAAATATTTTGGTG-3'
**IRE1KD-R(2694)**	Reverse primer for *IRE1* kinase domain site directed mutation	5'-CACCAAAATATTTTGTGGATTAATATTACGATGCACAATTTTTAA -3’
**IRE1ND-F(3456)**	Forward primer for *IRE1* kinase domain site directed mutation	5'-ATATAGTCCGGAAAAATTGATGCATCATTATAACGATATGCCAG-3'
**IRE1ND-R(3529)**	Reverse primer for *IRE1* kinase domain site directed mutation	5’-CTGGCATATCGTTATAATGATGCATCAATTTTTCCGGACTATAT-3’

**IRE1KD DET F(2319)**	For kinase mutation sequencing	5’-AATTCTTGGTTATGGTTCACATG-3’
**IRE1KD DET R(2921)**	For kinase mutation sequencing	5'- GAATCAGCAGATATTTCCCAT- 3'
**IRE1ND DET F(3215)**	For nuclease deletion sequencing	5'- GTCATGATCCTAGTCAACGTCC-3'
**IRE1KD DET R(2921)**	For nuclease deletion sequencing	5’-GGTATAACTATGTGTATGTG-3’
**HAC1SP(F)**	For *HAC1* intron splicing determination	5′-AGACGCTTTTAATTACCACACCA-3’
**HAC1SP(R)**	For *HAC1* splicing determination	5′-TCAAAGTCCAACTGAAATG-3’

Table S4: Oligonucleotides used for qPCR in this study.

**qACT1-F**	5’-GAAGCCCAATCCAAAAGA-3’
**qACT1-R**	5’-CTTCTGGAGCAACTCTCAATTC-3’
**qIRE1-F**	5’-AATTCTTGGTTATGGTTCACATG-3’
**qIRE1-R**	5’-AACTGCTACTGGTCGATTCTCAAA-3’
**qSEC61-F**	5'-GTCACAGAGACACTTCTGCTTACAA-3'
**qSEC61-R**	5'-TAGACGTACCAGAACCAAGAGTACC-3'
**qYSY6-F**	5'-ACACCTAAACAAAGAGCAGCTAATG-3'
**qYSY6-R**	5'-TTGCTCCACCACATACTAAGAA-3'
**qERD2-F**	5'-AACCAATCGATCCACCAATG-3'
**qERD-R**	5'-TGGAAGATGCAATGAAAACAA-3'
**qKAR2F**	5'CTGAAGATTACCTTGGCAAAAAAGT-3'
**qKAR2-R**	5' -TTAGTAGCTTGTCTTTGAGCATCGTT-3'
**qORF 19.2756-F**	5'-ATTGGATTTGGAATGCTTGG-3'
**qORF19.2756-R**	5'-TTCCGACAAATGTACCCACA-3'
**qERG24-F**	5'-ATGAAATCGTCAAAATTG-3'
**qERG24-R**	5'-GAGGTGATTGGTAATTGAC-3'
**qERG2-F**	5'-ATGAAGTTATTATTAGTAG-3'
**qERG2-R**	5'-AGTCAAATCAATCATAATGC-3'
**qERG11-F**	5'-ATGCTATTGTTGAAACTG-3'
**qERG11-R**	5'-AGAACCAAACCAAGGAATC-3'
**qHWP1-F**	5'-TGGTTCAGAACCATCCATGC-3'
**qHWP1-R**	5'-GGAATAGATGGTTGTGAACCAGC-3'
**qFET34-F**	5'-TGTTGGTGGATTTGTGTCACAGT-3'

**qFET34-R**	5'-CCCATCGACTTCAACGACAGT-3'
**qYWP1-F**	5'-TGCTGCTGCAAGTGCATTATT-3'
**qYWP1-R**	5'-TGTCCAAATCGACAACAGCAA-3'
**qSIT1-F**	5'-TCAACACTTGGATTTCCGGTAA-3'
**qSIT1-R**	5'-CCGATACCCCATTTCCATCTAG-3'
**qAMS1-F**	5'-TTCATATGCCTCCAGCGAATACT-3'
**qAMS1-R**	5'-CCAGCAGGGACATCACGAA-3'
**qSOD3-F**	5'-ACGGGTACAATGCCGCTATT-3'
**qSOD3-F**	5'-TTGAGATCCCTCTTGCCAACA-3'
**qECE1-F**	5'-CATGACTTCTGTTGCTTCTACCAAGA-3'
**qECE1-R**	5'-CAATCTGACGACGGCATTAGC-3'
